# Stem cell and stem cell-derived extracellular vesicle therapy for traumatic brain injury: mechanisms, engineering and translation

**DOI:** 10.3389/fnins.2026.1904590

**Published:** 2026-08-17

**Authors:** Shijun Bi, Dandan Gao, Kunyuan Zhu, Sanchuan Liu, Ligang Chen, Wei Jiang, Guobiao Liang

**Affiliations:** 1 Department of Neurosurgery, General Hospital of Northern Theater Command, Shenyang, China; 2 Graduate School, Dalian Medical University, Dalian, China

**Keywords:** extracellular vesicles, mesenchymal stem cells, neural stem cells, neuroinflammation, neuroregeneration, traumatic brain injury

## Abstract

Traumatic brain injury (TBI) remains a major unmet clinical challenge owing to its complex pathophysiology, profound heterogeneity, and limited regenerative capacity of the adult central nervous system (CNS). Existing therapeutic interventions are largely restricted to supportive management and fail to adequately address the secondary injury cascade characterized by persistent neuroinflammation, oxidative stress, vascular dysfunction, and progressive neurodegeneration. Against this backdrop, stem cell-based therapies have emerged as a rapidly expanding area of regenerative medicine with the potential to modulate multiple pathological processes simultaneously. In this Review, we summarize the therapeutic landscape of stem cell interventions for TBI, encompassing mesenchymal stem cells (MSCs), neural stem cells (NSCs), and cell-free derivatives including extracellular vesicles (EVs) and exosomes. We discuss the mechanistic basis of stem cell-mediated repair, focusing on immune microenvironment remodeling, endogenous neurogenesis, neurovascular restoration, apoptosis regulation, and neural circuit reconstruction. We further examine recent advances in therapeutic engineering, including biomaterial-assisted delivery systems, nanotechnology-enabled platforms, preconditioning paradigms, and multimodal combinatorial strategies designed to overcome limitations associated with cell survival, targeting, and functional integration. Finally, we evaluate the current status of clinical translation and highlight the principal biological, technical, and regulatory challenges that continue to impede therapeutic standardization and large-scale implementation. A deeper understanding of stem cell-microenvironment interactions, coupled with advances in bioengineering and rigorous clinical validation, will be essential for translating regenerative strategies into clinically effective therapies for TBI.

## 1 Introduction

TBI represents an increasingly severe global health crisis, with epidemiological studies reporting a staggering annual incidence of nearly 27 million acute cases worldwide (Saboori et al., 2024). This condition is a primary driver of both mortality and long-lasting physical or cognitive disabilities, impacting a vast population across all age groups and demographic profiles (Khazaal et al., 2025). A defining characteristic of TBI is its high clinical heterogeneity, where the initial mechanical insult sets in motion a diverse array of pathological events that often result in permanent neurological impairment (Borlongan and Rosi, 2022). Despite significant advancements in emergency medicine, there remains a notable lack of standardized therapeutic regimens capable of effectively combating the enduring long-term effects of the injury (Cui et al., 2022b). While current surgical and medical interventions are essential for acute survival, they are fundamentally limited in their ability to address the complex pathophysiology of secondary injury, which involves a cascade of neurotransmitter excitotoxicity and metabolic failure (Reis et al., 2017). Furthermore, the initial injury triggers a focal neuroinflammatory response; cell death within the damaged region induces a persistent immune reaction that leads to the further progression of tissue damage and potential cognitive decline (Dabrowska et al., 2019). Because existing therapeutic options frequently yield poor functional outcomes for TBI survivors, there is an urgent clinical mandate to develop regenerative strategies—most notably stem cell transplantation—that can move beyond palliative care to achieve genuine tissue restoration and functional repair (Zhou et al., 2019).

Recent bibliometric and visualized analyses demonstrate that stem cell interventions have evolved into a core research hotspot, reflecting a global academic trajectory focused on biological neurorestoration (Deng et al., 2024). Quantitative meta-analyses of preclinical data confirm that human stem cell transplantation significantly improves functional recovery, though the field increasingly demands standardized scientific rigor to bridge the translational gap (Chang et al., 2015). The prevailing therapeutic paradigm has shifted toward “paracrine regulation,” where MSCs target the pathological manifestations of TBI by secreting bioactive factors that mitigate oxidative stress and inflammation (Zhang et al., 2022). Genomic studies support this microenvironmental remodeling approach, showing that human mesenchymal stem cell treatment effectively normalizes cortical gene expression across multiple immune and signaling pathways that are otherwise disrupted by the initial trauma (Darkazalli et al., 2017). This evolution is further exemplified by the rise of “cell-free” therapeutics, particularly neural stem cell (NSC)-derived exosomes, which offer enhanced stability and blood-brain barrier (BBB) permeability compared to parent cells while maintaining potent neuroregulatory effects (Zhong et al., 2023). These collective developments establish a robust foundation for examining the specific cell types, mechanisms, and engineering strategies discussed in this review.

## 2 Cellular platforms for stem cell therapy in TBI

### 2.1 MSC-based therapeutic strategies

MSCs represent a cornerstone of regenerative medicine for TBI due to their multifaceted therapeutic potential. Current research has shifted from the traditional concept of cell replacement toward a focus on reducing secondary neurodegeneration and neuroinflammation through complex paracrine signaling (Das et al., 2019). Systematic investigations into the administration of these cells have demonstrated that intravenous infusion of MSCs, even when traversing the pulmonary microvasculature, can successfully facilitate the recovery of motor and cognitive functions (Harting et al., 2009). This therapeutic efficacy is primarily attributed to the ability of MSCs to alleviate the chronic neuroinflammation that typically exacerbates the secondary deterioration of brain structure and function (Bonsack et al., 2020).

Among the various sources, bone marrow-derived MSCs (BM-MSCs) are the most extensively characterized, providing a robust framework for clinical translation through their neurorestorative and anti-inflammatory properties (Cozene et al., 2021). Safety evaluations of these therapeutic populations emphasize their low immunogenicity and absence of tumorigenicity, with mechanisms involving the modulation of proinflammatory cytokines such as IL-6, IL-12, and TNF-α, while simultaneously promoting anti-inflammatory factors like IL-10 and TGF-β (Wang et al., 2022).

Complementing these findings, umbilical cord-derived MSCs (hUC-MSCs) have emerged as highly effective candidates, particularly in acute and developmental contexts. Preclinical models indicate that the intravenous administration of hUC-MSCs within a narrow window—specifically three hours post-injury—effectively protects brain architecture and preserves neurological integrity (Chen et al., 2020). In pediatric populations, autologous stem cell therapy is being explored to address the unique requirements of the developing brain, often focusing on sequestering systemic inflammatory responses linked to the spleen-brain axis (Dewan et al., 2018). Recent evidence further suggests that the neuroprotective benefits of hUC-MSCs are largely mediated by exosomes, which act via the lncRNA TUBB6/Nrf2 pathway to suppress TBI-induced inflammation and ferroptosis (Zhang et al., 2024). Together, these diverse MSC sources provide a versatile toolkit for addressing the heterogeneous pathology of brain trauma.

Adipose tissue-derived MSCs (ASCs) have gained increasing attention due to their unique paracrine profiles and therapeutic versatility. Investigations into ASC concentrated conditioned medium (ASC-CCM) demonstrate that these cells can modulate distal secondary complications, such as retinal damage following mild TBI, by regulating the expression of glutamate regulatory proteins and AQP4 (Jha et al., 2021). Human ASC treatments further provide robust cellular protection by reducing β-APP accumulation and suppressing proinflammatory cytokines like TNF-α, while their secretome alone is capable of preserving neural viability against mechanical strain in TBI models (Kappy et al., 2018).

The translation of MSC therapy into clinical practice has reached significant milestones, particularly with Wharton's Jelly-derived MSCs (WJ-MSCs). A Phase I clinical trial has confirmed the safety and efficiency of WJ-MSC administration in TBI patients, showing no serious adverse events across multiple delivery routes including intrathecal and intravenous injections (Kabatas et al., 2024). These findings are supported by pilot studies and clinical case reports indicating that WJ-MSC transplantation can effectively promote functional recovery and improve the neurological status of patients suffering from the chronic sequelae of brain trauma (Kabatas et al., 2020).

### 2.2 NSC–mediated neural repair

NSCs represent the most direct lineage-specific cell source for repairing the damaged CNS. In models of posttraumatic hyperexcitability, human embryonic stem cell (hESC)-derived NSCs demonstrate long-term survival and differentiate into neurons, astrocytes, and oligodendrocytes, providing a cellular basis for structural replacement (Beretta et al., 2017). The success of these transplants is highly dependent on the anatomical site of delivery. Subacute administration of human NSCs into the perilesional tissue, as opposed to the necrotic core, enhances engraftment and provides superior neuroprotection, which is critical for alleviating persistent motor deficits (Hu et al., 2020). To further validate clinical potential, human-induced pluripotent stem cell (hiPSC)-derived NSCs have been tested in pediatric large animal models. These cells effectively limit lesion expansion and promote tissue regeneration, facilitating significant functional recovery in the immature brain (Schantz et al., 2024). Beyond direct cell replacement, the paracrine activity of NSC populations is vital. Repeated intrathecal injections of peripheral nerve-derived stem cell spheroids, characterized by a Schwann cell-like phenotype, promote tissue repair by ensuring a sustained supply of essential neurotrophic factors (Shin et al., 2024). Clinical feasibility has been demonstrated through the transplantation of autologous mesenchymal stem cell-derived NSC-like cells in patients with severe TBI. This approach is safe and significantly elevates serum concentrations of NGF and BDNF, correlating with graded improvements in neurological function (Wang et al., 2017).

The development of hiPSC-derived neural cells facilitates the creation of individualized therapeutic platforms. Transplantation of these reprogrammed cells into the injured cortex demonstrates substantial potential for tissue replacement and functional restoration in standardized injury models (Furmanski et al., 2019). Beyond hiPSCs, human parthenogenetic NSCs offer a robust and standardized cell source, serving as potent mediators for multiple regenerative processes. These cells effectively bypass ethical concerns associated with embryonic sources while actively stimulating endogenous repair mechanisms following trauma (Lee et al., 2019). Advanced neural architectures, such as hESC-derived cerebral organoids, have also emerged as a novel strategy for addressing the complexities of brain injury. The integration of these three-dimensional organoid systems into injured sites has been shown to alleviate cognitive deficits and improve global brain recovery (Kim et al., 2022). Finally, the therapeutic efficacy of human neural stem/progenitor cells (hNS/PCs) is being optimized through synergistic engineering strategies. For instance, the co-administration of hNS/PCs with curcumin-loaded niosomal nanoparticles enhances recovery by creating a neuroprotective environment through the significant reduction of astrogliosis and brain edema (Narouiepour et al., 2022).

The therapeutic efficacy of NSC transplantation is profoundly dictated by the pathological and spatial context of the host environment. In scenarios involving posttraumatic hyperexcitability, hESC-derived NSCs transplanted into the injured cortex demonstrate the capacity to survive and differentiate into diverse neural lineages even when subjected to repeated electrical stimulation (Beretta et al., 2017). Beyond physiological states, the precise anatomical site of cell delivery serves as a critical determinant of regenerative success. Comparative analyses of intralesional vs. perilesional administration reveal that transplant location significantly influences engraftment dynamics, the progression of lesion expansion, and the ultimate degree of motor function amelioration (Hu et al., 2020). Furthermore, the interaction between NSCs and the host tissue is mediated by paracrine mechanisms within the injury microenvironment. The human NSC secretome plays a pivotal role in this process by relieving endoplasmic reticulum (ER) stress-induced apoptosis and promoting neuronal functional recovery, highlighting the necessity of modulating the biochemical milieu to optimize stem cell-based interventions (Ling et al., 2024).

### 2.3 Cell-free therapies: EVs and exosomes

EVs and exosomes derived from MSCs have emerged as pivotal “cell-free” therapeutic agents, offering a sophisticated alternative to traditional cell-based therapies by mitigating risks such as tumorigenicity and vascular occlusion. Clonal MSC-EVs significantly ameliorate TBI pathology by reducing cistauosis and neuronal apoptosis, thereby preserving tissue integrity and enhancing functional recovery in weight drop-induced injury models (Amini et al., 2023). The therapeutic efficacy of these derivatives is further exemplified by hUC-MSC-derived exosomes, which actively modulate the post-traumatic neuroinflammatory environment. These exosomes promote neurological function recovery specifically by suppressing the overactivation of microglia and astrocytes within the perilesional area (Cui et al., 2022a). Furthermore, the utility of MSC-EVs extends to systemic protection against secondary injury processes. In models of inflammation-induced brain injury, the administration of these vesicles effectively ameliorates the systemic inflammatory response and protects the developing brain from white matter damage, demonstrating their robust capacity for neuroprotection and microenvironmental stabilization (Drommelschmidt et al., 2017).

EVs have emerged as a pivotal cell-free therapeutic strategy, leveraging their inherent ability to cross the BBB and modulate the complex pathological environment of TBI (Xiong et al., 2024). In clinically relevant porcine models involving both TBI and hemorrhagic shock, MSC-derived exosomes have demonstrated significant neuroprotective effects by improving long-term neurological outcomes (Williams et al., 2019). These benefits are largely attributed to the modulation of the brain transcriptome, where a single early dose of EVs can initiate protective genomic shifts (Bayat Tork et al., 2025). Systemic administration of these vesicles in rodent models further facilitates functional recovery, exhibiting a distinct dose-response relationship and a viable therapeutic window for intervention (Zhang et al., 2020b).

**Figure 1:**
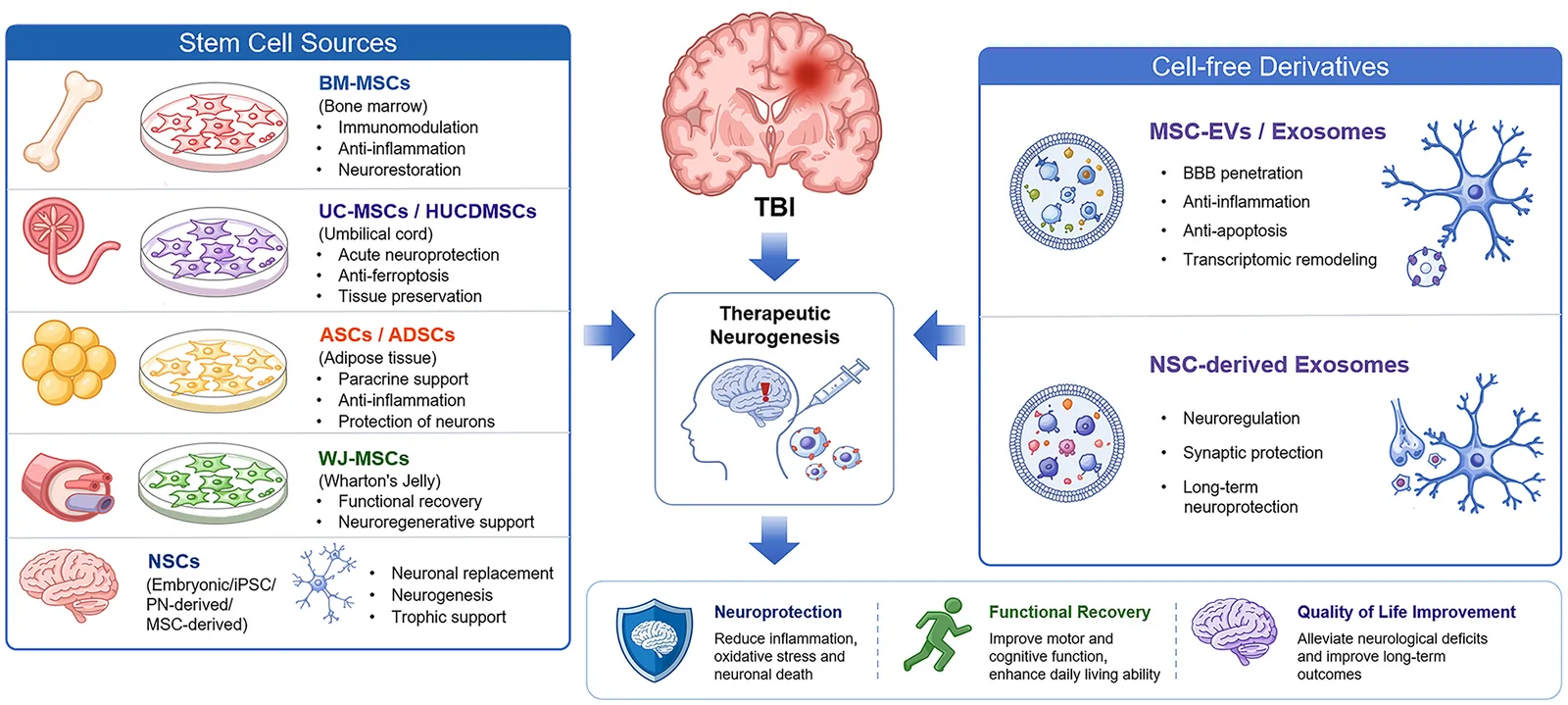
Therapeutic landscape of stem cell-based interventions for traumatic brain injury.

To optimize the regenerative microenvironment, advanced delivery systems like electrospun nanofibrous platforms are now employed to provide sustained local release of dual exosomes derived from both MSCs and NSCs (Li et al., 2024). Specifically, NSC-derived exosomes offer unique advantages in neuroregulation and tissue repair due to their high stability and low immunogenicity compared to direct cell transplantation (Zhong et al., 2023). Figure 1 provides a compact overview of the major stem cell populations, representative advantages, and principal therapeutic outputs discussed in this section.

Although extracellular vesicles and exosomes have demonstrated considerable therapeutic potential in preclinical studies, several important challenges continue to impede their clinical translation. Variability in donor cell sources, cell culture conditions, and isolation procedures frequently leads to substantial batch-to-batch differences in vesicle composition and biological activity, making therapeutic outcomes difficult to reproduce. In addition, the relatively low yield and limited scalability of current isolation techniques remain significant barriers to large-scale manufacturing. Another important issue is the lack of uniform standards for vesicle characterization. Inconsistent reporting of particle size, surface markers, purity, and biological activity complicates comparisons across studies and reduces the reproducibility of experimental findings. These methodological limitations should be taken into account when evaluating the promising therapeutic effects reported in experimental models.

For a direct comparison of the major cell sources and cell-free derivatives discussed above, Table 1 compiles their origins, therapeutic features, strengths, and key translational limitations.

**Table 1:** Stem cell sources and cell-free derivatives for traumatic brain injury therapy.

Cell source/ derivative	Representative subtype or origin	Principal therapeutic actions in TBI	Major advantages	Key translational limitations	References
BM-MSCs	Bone marrow-derived mesenchymal stem cells	Immunomodulation, attenuation of neuroinflammation, neurorestoration, paracrine regulation	Most extensively characterized MSC population; relatively mature translational foundation; low immunogenicity	Therapeutic heterogeneity; efficacy influenced by timing, dosage, and delivery route	Harting et al., 2009; Bonsack et al., 2020; Cozene et al., 2021; Wang et al., 2022
UC-MSCs/HUCDMSCs	Umbilical cord-derived MSCs	Acute neuroprotection, preservation of brain architecture, anti-ferroptosis activity	Strong proliferative potential; suitable for acute and developmental TBI settings	Therapeutic window remains incompletely standardized	Chen et al., 2020; Dewan et al., 2018; Zhang et al., 2024
ASCs/ADSCs	Adipose-derived mesenchymal stem cells	Suppression of inflammatory cytokines, protection against secondary degeneration, paracrine neuroprotection	Easily accessible source with abundant yield	Comparatively limited long-term translational evidence	Jha et al., 2021; Kappy et al., 2018; Tang et al., 2023
WJ-MSCs	Wharton's Jelly-derived MSCs	Functional recovery and neuroregenerative support	Favorable safety profile demonstrated in phase I studies	Large-scale clinical validation still lacking	Kabatas et al., 2024, 2020
NSCs	Embryonic-, iPSC-, parthenogenetic-, or peripheral nerve-derived NSCs	Neuronal replacement, trophic support, endogenous neurogenesis stimulation	Strong lineage specificity for CNS repair	Engraftment and survival highly dependent on injury microenvironment	Beretta et al., 2017; Hu et al., 2020; Schantz et al., 2024; Wang et al., 2017; Furmanski et al., 2019; Lee et al., 2019; Kim et al., 2022
MSC-EVs/exosomes	Extracellular vesicles derived from MSCs	BBB penetration, anti-inflammatory signaling, apoptosis inhibition, transcriptomic remodeling	Lower tumorigenic and vascular occlusion risks than cell transplantation	Manufacturing standardization and cargo consistency remain unresolved	Amini et al., 2023; Cui et al., 2022a; Drommelschmidt et al., 2017; Williams et al., 2019; Xiong et al., 2024; Zhang et al., 2020b
NSC-derived exosomes	Extracellular vesicles derived from NSCs	Synaptic protection, neuroregulation, long-term neuroprotection	High stability and low immunogenicity	Early-stage translational development	Zhong et al., 2023; Kodali et al., 2023

## 3 Mechanisms underlying stem cell-mediated repair in TBI

### 3.1 Anti-inflammatory and immune remodeling

Stem cell therapies and their derivatives, particularly EVs, play a pivotal role in modulating the post-TBI inflammatory cascade by reconfiguring the response of microglia and astrocytes. hiPSC-derived microglia have demonstrated the capacity to release significant levels of pro-inflammatory cytokines such as IL-6 and TNF in response to the injury environment, a process that can be strategically targeted by stem cell interventions to limit damage amplification (Alam et al., 2023). For instance, delivery systems utilizing brain-derived tissue-specific bioinks have been shown to effectively carry NSCs into the lesion site, where they actively suppress the activation and proliferation of GFAP-positive reactive astrocytes and Iba-1-positive microglia (Bae et al., 2021). Furthermore, treatment with human MSCs has been found to normalize cortical gene expression, specifically downregulating pathways associated with cytokine-cytokine receptor interactions and the JAK-STAT signaling pathway, which are typically overactivated following TBI (Darkazalli et al., 2017). In addition to direct cell transplantation, MSC-EVs offer a potent cell-free alternative; systemic administration of these vesicles can significantly reduce the infiltration of CD45-positive leukocytes and decrease the expression of pro-inflammatory markers like COX2 and IL-1β in the injured brain (Drommelschmidt et al., 2017). Mechanistically, exosomes derived from bone marrow stromal cells can target DAB2IP to induce microglial autophagy, thereby creating a more permissive environment for the survival and integration of subsequent NSC grafts (Yuan et al., 2020).

Stem cells and their derivatives demonstrate substantial capabilities in remodeling the immune microenvironment following TBI. Systemic transplantation of MSCs significantly reduces the density of local microglia/macrophages and peripherally infiltrating leukocytes within the damaged brain tissue. Concurrently, it attenuates the release of pro-inflammatory cytokines and elevates anti-inflammatory cytokine levels, a process partially mediated by the upregulation of TSG-6 expression and the subsequent inhibition of the NF-κB signaling pathway (Zhang et al., 2013). Beyond direct cellular replacement, MSCs and their EVs suppress excessive astrocyte activation and drive microglial polarization from the pro-inflammatory M1 phenotype toward the anti-inflammatory M2 phenotype, thereby restoring the local immune microenvironment (Zhang et al., 2022). Investigations into adipose-derived stem cell exosomes (ADSC-Exos) further confirm their efficacy in downregulating the secretion of pro-inflammatory mediators, including IL-1β, IL-6, and TNF-α, by inhibiting the activation of the microglial NLRP3 inflammasome, which consequently mitigates neuroinflammation (Tang et al., 2023). Within the neuroinflammatory cascade, studies utilizing hESC-derived neuron models verify that neurons exhibit complex secondary cytokine responses upon exposure to inflammatory factors such as TNF, underscoring the critical necessity of timely inflammatory microenvironment suppression for neuronal survival (Thelin et al., 2018). Among adjuvant physical interventions, the application of electrical stimulation during the acute injury phase not only increases the population of NSCs and Nestin-positive astrocytes in the lesioned area but also significantly enriches the CD206-positive anti-inflammatory microglial phenotype (Park et al., 2021).

Importantly, the post-TBI neuroinflammatory microenvironment is heavily driven by a sophisticated molecular crosstalk between different programmed cell death pathways, where NLRP3 inflammasome-mediated pyroptosis and subsequent necroptosis form a vicious pathological amplification loop. Paracrine factors derived from MSCs play a paramount role in interrupting this detrimental cascade (Bonsack et al., 2020). Specifically, ADSC-Exos have been shown to drastically suppress the activation of the microglial NLRP3 inflammasome and caspase-1, thereby halting the cleavage of gasdermin D and downstream pyroptosis (Tang et al., 2023). By fundamentally blocking NLRP3-mediated pyroptosis, MSC paracrine actions efficiently curtail the extracellular release of cellular damage-associated molecular patterns (DAMPs) and pro-inflammatory components (Cui et al., 2022a). Crucially, because these released DAMPs serve as primary upstream triggers that induce RIPK1/RIPK3-dependent necroptosis in neighboring neural cells, the MSC-mediated paracrine profiling successfully severs the crosstalk between pyroptosis and necroptosis, ultimately stabilizing the post-traumatic immune milieu (Dabrowska et al., 2019).

Importantly, engineered EVs exert potent neuroprotection by preserving mitochondrial homeostasis, acting as a critical metabolic hub to block interconnected regulated cell death cascades (Li et al., 2024). Traumatic brain injury triggers profound mitochondrial dysfunction, accelerating reactive oxygen species (ROS) accumulation and defective mitophagy that fuel parallel death pathways. To counteract this, engineered EVs can robustly restore mitochondrial structural and functional integrity. Specifically, modified MSC-derived EVs upregulate the PINK1/Parkin pathway to activate beneficial mitophagy, effectively clearing damaged mitochondria and halting downstream programmed cell death (Zhang et al., 2023a). Furthermore, engineered EVs activate the lncRNA TUBB6/Nrf2 signaling axis, enhancing mitochondrial antioxidant defenses and suppressing catastrophic lipid peroxidation (Zhang et al., 2024). By stabilizing mitochondrial metabolic activity and mitigating oxidative stress, these engineered nano-vesicles successfully sever the pathological link between mitochondrial collapse and synchronized cell death cascades (Li et al., 2024).

### 3.2 Anti-apoptotic and cell survival

Following traumatic injury, engrafted NSCs have been shown to significantly inhibit neuronal apoptosis through the upregulation of Bcl-xL, a key anti-apoptotic protein, which facilitates the recovery of motor and equilibrium functions (Pang et al., 2017). These cellular interventions are increasingly supplemented by bioengineering approaches, such as the use of BdECM bioinks, which provide a biomimetic microenvironment that enhances cell survival and suppresses the overactivation of glial cells (Bae et al., 2021). This paracrine-dominant perspective is further supported by evidence that the NSC secretome can independently improve neuronal function by relieving ER stress and inhibiting apoptosis without the need for physical cell integration (Ling et al., 2024). At the molecular level, hUC-MSC-derived exosomes suppress programmed cell death within the injured tissue by activating the PINK1/Parkin-mediated mitophagy pathway (Zhang et al., 2023a). Furthermore, non-invasive delivery methods, such as intranasal administration, have proven effective in easing the decline of neurogenesis and preventing synapse loss via the BDNF-ERK-CREB signaling cascade (Kodali et al., 2023).

The progressive loss of neural elements following TBI is fundamentally driven by a complex interconnected network of programmed cell death cascades rather than isolated lethal pathways. In the post-traumatic microenvironment, severe secondary injury triggers critical cellular stresses, among which ER stress stands out as a primary initiator of neuronal apoptosis. Sustained ER stress drives irreversible cellular damage, directly promoting the cleavage of pro-apoptotic executioners, notably Caspase-3, and leading to catastrophic neuronal loss. Accumulating evidence demonstrates that stem cells and their derived EVs exert potent neuroprotective effects by systematically dampening this cellular stress response; specifically, NSC secretome delivery significantly relieves ER stress-induced injury in traumatized brain tissues, effectively downregulating downstream cleaved Caspase-3 expression and protecting vulnerable neurons (Ling et al., 2024). Furthermore, this anti-apoptotic signaling is robustly reinforced by the survival-promoting machinery within host neural cells. NSC transplantation has been shown to induce a dramatic upregulation of the anti-apoptotic protein Bcl-xL within the lesioned cortex, which stabilizes the outer mitochondrial membrane and facilitates substantial recovery of neurological functions (Pang et al., 2017).

Crucially, the neurotrauma field has shifted from classical single-pathway death models toward evaluating comprehensive multi-pathway programmed cell death networks, particularly PANoptosis—a highly integrated, pro-inflammatory cell death pathway featuring the molecular co-execution of apoptosis, pyroptosis, and necroptosis. Driven by post-TBI mitochondrial collapse and neuroinflammatory propagation, PANoptosis forms a destructive amplification loop that accelerates tissue degradation. To interrupt this interconnected death cascade, stem cell-based therapies focus on the precise preservation of mitochondrial quality control and homeostasis. Mesenchymal stem cell (MSC)-derived exosomes effectively provide neuroprotection by robustly activating PINK1/Parkin-mediated mitophagy, which ensures the timely clearance of damaged, dysfunctional mitochondria and mitigates ROS accumulation (Zhang et al., 2023a). By incorporating this mitochondrial defense, MSC transplantation—especially when integrated with synergistic physical interventions—robustly modulates microglial polarization, dampens neuroinflammatory infiltration, and successfully mitigates cerebral injury-induced neuronal PANoptosis (Cheng et al., 2024). Alternatively, when engineered or combined with other advanced natural products such as curcumin, primed stem cells effectively prevent the execution of parallel programmed death pathways, creating a multi-dimensional defense network that ensures long-term cell survival and neural tissue remodeling (Lan et al., 2024).

### 3.3 Anti-oxidative stress and anti-ferroptosis

The delivery of stem cell therapies actively counters the intense oxidative stress and metabolic dysregulation characterizing secondary neurotrauma. Exogenous NSC transplantation combined with the antioxidant recombinant protein PEP-1-SOD1 effectively mitigates oxidative stress injury and upregulates AQP4 expression to accelerate neurological functional recovery in rats (Jia et al., 2018). The application of recombinant PEP-1-SOD1 serves as a potent antioxidant shield, protecting transplanted NSCs from oxidative stress and significantly boosting motor and sensory recovery (Jia et al., 2018). This microenvironmental stabilization is further enhanced by paracrine mechanisms, specifically through hUC-MSC-derived exosomes that activate the lncRNA TUBB6/Nrf2 signaling pathway to suppress TBI-induced inflammation and ferroptosis, thereby maintaining neural integrity (Zhang et al., 2024).

In addition to suppressing ferroptotic lipid peroxidation, advanced stem cell interventions are now recognized to mitigate cuproptosis, a recently identified form of regulated cell death driven by intracellular copper overload. TBI disrupts copper homeostasis, leading to the direct binding of copper to lipoylated components of the tricarboxylic acid (TCA) cycle, which induces proteotoxic stress, mitochondrial enzyme aggregation, and respiratory collapse. Engineered EVs effectively intervene in this cascade by stabilizing mitochondrial metabolic flux and promoting copper-transporting homeostatic mechanisms, shielding neural cells from synchronized ferroptotic and cuproptotic challenges.

### 3.4 Endogenous neurogenesis activation

TBI serves as a primary stimulus for innate repair mechanisms, notably triggering the proliferation of NSCs in the adult hippocampal subgranular zone via the rapid activation of the mTORC1 signaling pathway (Wang et al., 2016). The endogenous neurogenic response is subject to complex regulatory checkpoints; for instance, TLR2 has been identified as a critical factor that attenuates TBI-induced NSC proliferation in the dentate gyrus (Zhang et al., 2020a). Molecular modulation can further unlock regenerative capacity, as seen with the downregulation of miR-124-3p, which activates NSCs in the SVZ by enhancing the efficiency of BDNF downstream signaling (Kang et al., 2022). The downregulation of the microRNA miR-124-3p enhances the downstream signaling pathways of brain-derived neurotrophic factor (BDNF), including the PI3K/Akt and Ras pathways, thereby efficiently promoting the activation and proliferation of NSCs in the subventricular zone (SVZ) (Kang et al., 2022). In murine models, the upregulated expression of Toll-like receptor 4 (TLR4) in the hippocampus is closely correlated with the proliferation of endogenous NSCs post-TBI, implying a potential role for innate immune receptors in neurogenesis (Ye et al., 2014). Enhancement strategies such as docosahexaenoic acid (DHA) supplementation further improve the therapeutic potential and integration of transplanted neonatal NSCs (Ghazale et al., 2018).

### 3.5 Migration and homing

For successful structural restoration, the migration of these progenitors to the lesion site is essential. This directed movement is facilitated by neuronal expression of SCF, which serves as a chemoattractant to recruit NSPCs to injured regions through c-kit receptor activation (Sun et al., 2004). The cellular niche also significantly influences these outcomes; neonatal astrocytes exhibit a superior ability to drive NSC proliferation by utilizing TNC to bind EGFR and activate the PI3K-AKT pathway (Dai et al., 2019). In the context of bioengineering, the use of functionalized nano-scaffolds containing stromal cell-derived factor 1 (SDF-1) motifs—designed with a Young's modulus of 3.21 kPa to match neural tissue—has been shown to enhance NSC proliferation and migration while promoting synaptogenesis (Bayat Tork et al., 2025).

Furthermore, beyond growth factors, hyperbaric oxygen (HBO) therapy serves as a critical adjunct by creating a favorable metabolic state that activates the VEGF/ERK signaling pathway, thereby stimulating the proliferation and targeted migration of NSCs toward the lesion area (Yang et al., 2017). The co-administration of hematopoietic growth factors, specifically SCF and G-CSF, provides a powerful stimulus for reducing long-term neurodegeneration and promoting the reorganization of neural networks after severe TBI (He et al., 2020).

### 3.6 Angiogenesis and vascular remodeling

Stem cell therapy facilitates functional recovery following TBI through a multi-faceted approach involving the orchestration of angiogenesis and the stabilization of the host microenvironment. The systemic administration of hUC-MSCs has been demonstrated to effectively preserve brain architecture by attenuating the release of pro-inflammatory cytokines and concurrently upregulating the expression of VEGF, which is fundamental for re-establishing vascular networks and providing nutritional support to the damaged cortex (Chen et al., 2020). To overcome structural delivery limitations, combining exosome therapy with engineered constructs has shown a strong capacity to stimulate extensive functional vascularization and tissue re-perfusion in canine models (Liu et al., 2022).

### 3.7 Synaptic remodeling and neuroplasticity

NSC transplantation facilitates robust functional recovery after TBI, as evidenced by significant improvements in motor coordination and neurological severity scores. This regenerative effect is largely attributed to BDNF-mediated neuroplasticity, where transplanted cells drive the upregulation of Synaptophysin to support the reconstruction of functional neural networks (Xiong et al., 2018). To overcome the challenges of the harsh injury site, bioengineered delivery systems like chondroitin sulfate glycosaminoglycan (CS-GAG) matrices are utilized to enrich FGF2. These matrices sustain the expression of Sox1 and Nestin, maintaining the transplanted NSCs in an undifferentiated state that is essential for long-term neuroprotection and tissue repair (Betancur et al., 2017).

Furthermore, stem cell-derived cell-free therapies provide critical protection against chronic neurodegeneration. Intranasal delivery of human MSC-EVs has been shown to mitigate the decline of endogenous neurogenesis in the subgranular zone during the chronic phase. By activating the BDNF-ERK-CREB signaling pathway, these vesicles prevent the loss of PSD95-positive synapses and preserve the structural integrity of hippocampal circuits long after the initial trauma (Kodali et al., 2023).

### 3.8 Remyelination and white matter repair

The protection and reconstruction of damaged white matter tracts represent an essential mechanical component of the long-term functional recovery driven by stem cell interventions. Stem cell-derived extracellular vesicles offer a robust cell-free strategy for protecting structural networks; specifically, MSC-EVs have demonstrated the capacity to prevent neuronal loss and protect white matter integrity, even in the presence of severe inflammatory stimuli (Drommelschmidt et al., 2017). In the chronic phase of severe injury, targeted dual-factor protocols can be deployed to further optimize structural outcomes. The administration of specialized growth factors, such as the combination of SCF and G-CSF, is particularly effective in the chronic phase of injury, where it supports remyelination and the repair of white matter (Qiu et al., 2023).

In neonatal or developmental brain injury, multi-factor protocols involving SCF, G-CSF, and FLT3L have been shown to provide sustained neuroprotection and improve behavioral outcomes across short- and long-term intervals (Posod et al., 2019). To optimize clinical translation, the integration of cellular grafts with physical therapies has shown substantial benefits; clinical-grade UC-MSC transplantation, when synchronized with HBO sessions, results in a synergistic increase in axon-like structures and superior neurological recovery compared to either treatment used in isolation (Zhou et al., 2016).

**Figure 2:**
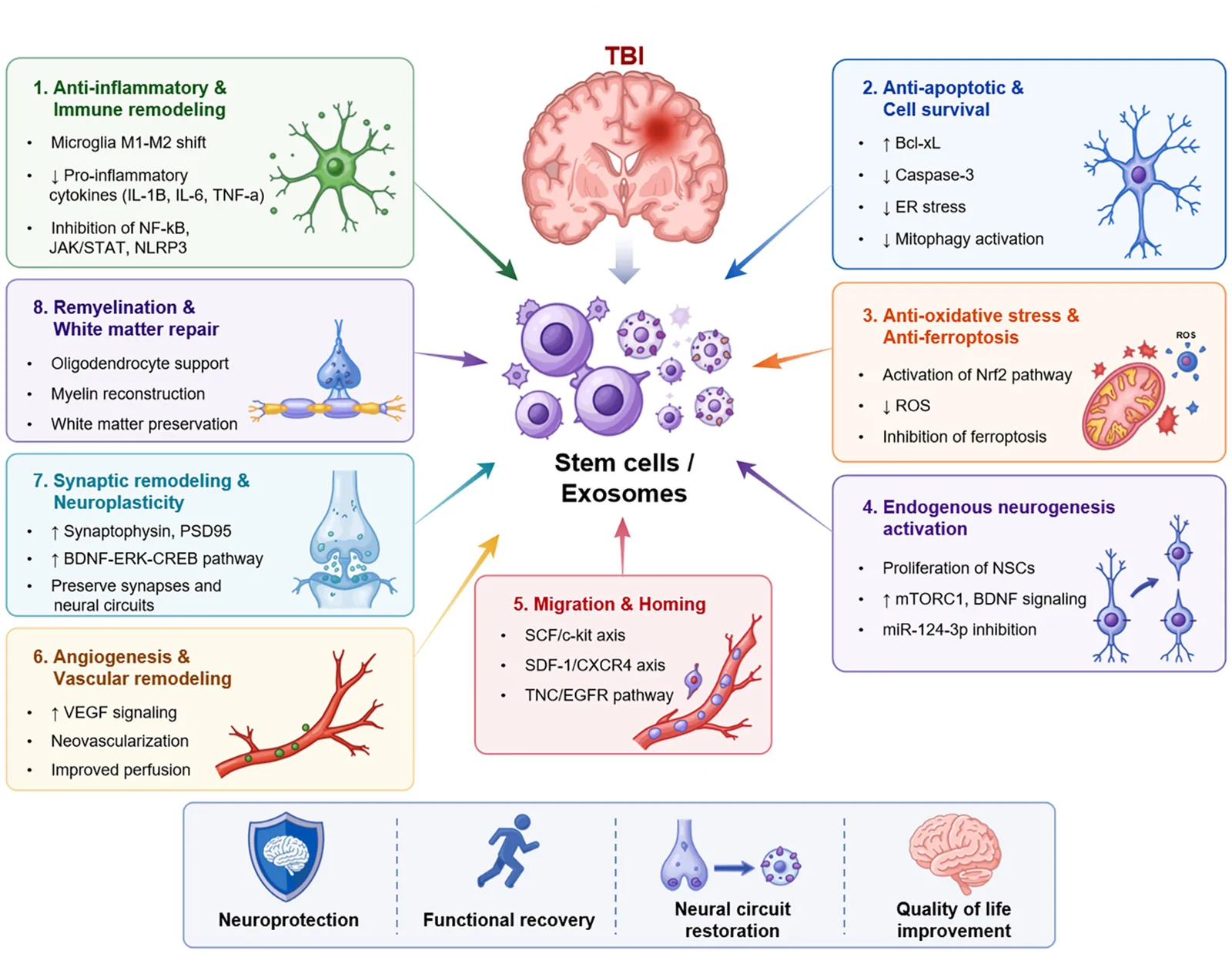
Mechanistic framework underlying stem cell-mediated neurorepair in traumatic brain injury.

Collectively, these studies show that the benefits of stem cell therapy arise from coordinated actions on inflammation, survival, oxidative stress, endogenous repair, and circuit remodeling. Figure 2 summarizes these convergent mechanisms in a single visual framework. Given the overlap among these signaling events, Table 2 organizes the principal mechanisms into a pathway-oriented summary that links each mechanistic axis to representative molecular mediators and functional outcomes.

**Table 2:** Mechanistic landscape of stem cell-based therapies in traumatic brain injury.

Mechanistic category	Representative pathways or mediators	Biological consequences in TBI	Representative outcomes	References
Anti-inflammatory and immune remodeling	NF-κB, JAK/STAT, NLRP3 inflammasome, microglial M1 → M2 polarization	Suppression of excessive neuroinflammation and immune infiltration	Reduced IL-1β, IL-6, TNF-α, GFAP, and Iba-1 expression	Zhang et al., 2022; Darkazalli et al., 2017; Dewan et al., 2018; Bae et al., 2021; Zhang et al., 2013; Tang et al., 2023
Anti-apoptotic regulation	Bcl-xL upregulation, ER stress inhibition, mitophagy activation	Prevention of neuronal death and preservation of tissue integrity	Reduced apoptosis and improved neurological recovery	Ling et al., 2024; Amini et al., 2023; Drommelschmidt et al., 2017; Zhang et al., 2023a; Pang et al., 2017
Anti-oxidative stress and anti-ferroptosis effects	TUBB6/Nrf2 signaling, antioxidant enzyme delivery	Reduction of oxidative injury and ferroptotic damage	Attenuation of edema and enhanced graft survival	Zhang et al., 2024; Jia et al., 2018
Endogenous neurogenesis activation	mTORC1 activation, miR-124-3p inhibition, BDNF signaling	Promotion of NSC proliferation and neuroplasticity	Increased endogenous progenitor activation and improved functional outcomes	Ye et al., 2014; Kang et al., 2022; Wang et al., 2016; Xiong et al., 2018
Stem cell migration and homing	SCF/c-kit axis, SDF-1 signaling, TNC/EGFR pathway	Enhanced recruitment of endogenous or transplanted stem cells	Improved lesion targeting and engraftment efficiency	Bayat Tork et al., 2025; Sun et al., 2004; Dai et al., 2019; Zamproni et al., 2017
Angiogenesis and neurovascular remodeling	VEGF signaling, hypoxia-preconditioned MSC-Exos	Restoration of vascular support and tissue perfusion	Enhanced vascularization and neural regeneration	Chen et al., 2020; Liu et al., 2022; Yang et al., 2017; Zhou et al., 2016
Synaptic remodeling and neuroplasticity	BDNF-ERK-CREB signaling, Synaptophysin, PSD95 preservation	Reconstruction of neural circuits and synaptic maintenance	Improved cognitive and motor recovery	Zhong et al., 2023; Kodali et al., 2023; Xiong et al., 2018
Remyelination and white matter repair	SCF + G-CSF combination therapy, MSC-EVs	Promotion of oligodendrocyte regeneration and axonal integrity	Improved white matter preservation and chronic recovery	Drommelschmidt et al., 2017; He et al., 2020; Qiu et al., 2023

## 4 Engineering strategies to enhance stem cell therapy

### 4.1 Biomaterials and advanced delivery platforms

The engineering of sophisticated delivery systems has transitioned from simple cell suspension injections toward the creation of “biomimetic microenvironments” that facilitate tissue integration. Brain-derived decellularized extracellular matrix (BdECM) bioinks represent a major advancement in this direction, as they provide a tissue-specific substrate that promotes the differentiation of NSCs into functional neurons while simultaneously suppressing the local inflammatory response following injury (Bae et al., 2021).

To further optimize the biochemical and mechanical cues of the injury niche, innovative nano-scaffolds incorporating SDF-1 motifs have been developed. These scaffolds are engineered with a Young's modulus of approximately 3.21 kPa to match the elasticity of native neural tissue, thereby enhancing NSC proliferation and synaptogenesis (Bayat Tork et al., 2025). The biochemical signaling within these matrices is equally critical; for instance, CS-GAG matrices enriched with FGF2 have been shown to maintain the undifferentiated state of NSCs and offer significant neuroprotection during the subacute phase of TBI (Betancur et al., 2017). This microenvironmental regulation is further supported by the inclusion of specific matricellular proteins, such as Tenascin-C (TNC), which binds to the epidermal growth factor receptor (EGFR) on NSCs to promote their proliferation through the PI3K-AKT signaling pathway (Dai et al., 2019). In the context of localized delivery, hydrogel-based delivery sheets for NSC spheroids have proven effective in reducing cortical lesion volume and reversing cognitive impairment in mild TBI models (Kim et al., 2023). Complementary research using imidazole-functionalized GelMA hydrogels loaded with human amnion-derived MSCs and SDF-1a has demonstrated a synergistic effect in promoting stem cell differentiation and repairing focal brain damage (Zheng et al., 2021). Beyond direct cell replacement, the protection of the overall brain architecture is a primary goal, with evidence suggesting that the intravenous delivery of hUC-MSCs can effectively preserve neurological function and structural integrity (Chen et al., 2020). However, clinical translation still faces significant hurdles regarding cell survival and delivery, prompting the use of encapsulated cell biodelivery systems—such as MSCs engineered to secrete GLP-1—which provide a protected reservoir for sustained therapeutic factor release (Heile and Brinker, 2011).

Advanced fabrication techniques such as electrospinning and 3D printing have further expanded the possibilities for localized, sustained intervention. These exosomal cargoes exert their effects through specific molecular mechanisms, such as the lncRNA TUBB6/Nrf2 pathway, which suppresses inflammation and ferroptosis in the injured brain (Zhang et al., 2024). Furthermore, composite tissue-engineered implants fabricated via low-temperature extrusion 3D printing preserve the bioactivity of encapsulated exosomes while allowing custom spatial conformality, providing high therapeutic scalability for neural tissue repair (Liu et al., 2022).

### 4.2 Nanotechnology-assisted therapeutic delivery

Advanced nanotechnology provides a robust framework to enhance delivery precision and cargo stability in TBI, effectively overcoming biological barriers such as rapid enzymatic degradation and poor tissue penetration. The localized application of SDF-1 loaded PLGA nanoparticles has been shown to effectively stimulate the recruitment of endogenous NSCs to the site of injury, providing a sustained release profile that facilitates long-term tissue repair (Zamproni et al., 2017). To address the hostile post-traumatic environment, the development of nanodendriplexes designed to deliver shRNA plasmids against CCL20 and CCR6 has proven effective in downregulating pro-inflammatory cytokines; such targeted molecular intervention primes the brain tissue, thereby significantly augmenting the neuroprotective efficacy of subsequent hMSC transplantation (Mayilsamy et al., 2020).

The evolution toward multimodal delivery systems integrates cellular components with pharmacological agents and biomaterials to maximize regenerative synergy. Combining hNS/PC transplantation with the oral administration of curcumin-loaded niosomal nanoparticles demonstrates substantial therapeutic benefits, including reduced astrogliosis and enhanced functional recovery through the synergistic modulation of the oxidative and inflammatory niche (Narouiepour et al., 2022). Furthermore, sophisticated tissue engineering platforms are increasingly employed to deliver stem cell derivatives in a controlled, localized manner. An electrospun nanofibrous scaffold functionalized with polydopamine has been successfully utilized for the sustained delivery of dual exosomes derived from both MSCs and NSCs (Li et al., 2024). This dual-exosome system effectively promotes neurovascular remodeling, providing a comprehensive repair mechanism that targets multiple facets of TBI pathology while maintaining a high degree of clinical translatability.

### 4.3 Preconditioning and combination therapeutic approaches

To overcome the hostile post-injury microenvironment, preconditioning and multimodal combination strategies have been increasingly utilized to enhance the efficacy of stem cell-based therapies. For instance, DHA has been identified as a potent agent that significantly augments the therapeutic potential of neonatal NSC transplantation following TBI, facilitating improved functional recovery and neural differentiation (Ghazale et al., 2018). In addition to pharmacological preconditioning, physical interventions such as HBO therapy serve as vital adjuncts. HBO has been shown to stimulate the proliferation and targeted migration of NSCs to the lesion area by activating the VEGF/VEGFR2 and Raf-1/MEK/ERK signaling cascades (Yang et al., 2017). The synergistic application of HBO with UC-MSC transplantation demonstrates superior therapeutic outcomes compared to monotherapy, specifically by promoting the formation of axon-like structures and enhancing overall tissue repair (Zhou et al., 2016). However, the integration of these interventions requires precise temporal management and a nuanced understanding of their interactions. While acute hypothermia and delayed MSC therapy independently offer protection against motor and cognitive deficits, their combined application may lead to complex interactions that increase neuro-inflammatory responses and potentially diminish the sub-acute neuroprotective benefits observed in single-modality treatments (Herz et al., 2018). This paradoxical antagonism is fundamentally dictated by the therapeutic window and the evolving host microenvironment. Acute physical adjuvants, such as hypothermia, are designed to profoundly suppress early inflammatory cascades and minimize immediate tissue necrosis. However, delayed cell grafts, particularly MSCs, frequently require a specific threshold of early inflammatory cues (e.g., TNF-α and IL-1β) to undergo “licensing”—the physiological priming essential for activating their full immunomodulatory, paracrine, and anti-inflammatory capacities (Herz et al., 2018). When acute physical interventions prematurely or excessively dampen these early alarmins, a chronological mismatch occurs. Consequently, the subsequently transplanted cells fail to receive adequate microenvironmental activation, which can paradoxically trigger a secondary, destructive rebound of pro-inflammatory cytokines during the sub-acute phase. This indicates that physical adjuvants like hypothermia or HBO can act either synergistically or antagonistically with cell grafts depending strictly on whether their application windows respect the biphasic pathophysiology of brain injury. To achieve true synergy, the suppression of acute-phase toxicity must be rigorously balanced against the preservation of sub-acute microenvironmental cues necessary for graft survival and function. These findings highlight the importance of optimizing the host environment to maximize the regenerative capacity of transplanted or endogenous cells.

The synergistic administration of stem cell factor (SCF) and granulocyte colony-stimulating factor (G-CSF) has emerged as a robust strategy to ameliorate widespread neurodegeneration and facilitate the reorganization of neural networks, leading to improved cognitive outcomes after severe TBI (He et al., 2020). This combination therapy also plays a critical role in the chronic phase by promoting remyelination, which is essential for restoring white matter integrity and long-term functional recovery (Qiu et al., 2023). In neonatal models of brain injury, the integration of hematopoietic growth factors including G-CSF, SCF, and FLT3L has been investigated to provide sustained neuroprotection across short-, mid-, and long-term recovery periods (Posod et al., 2019). Beyond growth factors, the modulation of microRNAs offers a precise tool for enhancing endogenous repair; specifically, inhibiting miR-124-3p activates NSCs in the SVZ by amplifying BDNF signaling cascades (Kang et al., 2022). Additionally, the interplay between innate immunity and regeneration is highlighted by the association between TLR4 expression and NSC proliferation in the hippocampus, indicating that the immune microenvironment can be harnessed to drive neurogenesis (Ye et al., 2014).

Accordingly, Table 3 outlines the engineering and combination strategies that most effectively enhance stem cell therapy for TBI, with emphasis on biomaterials, nanotechnology, preconditioning, and adjunctive interventions that improve translational feasibility.

**Table 3:** Engineering and combination strategies for enhancing stem cell therapy in traumatic brain injury.

Strategy category	Representative platform or intervention	Mechanistic rationale	Therapeutic benefits	Translational significance	References
Biomimetic scaffolds and hydrogels	BdECM bioinks, CS-GAG matrices, GelMA hydrogels	Reconstruction of a permissive regenerative niche	Improved NSC survival, differentiation, and tissue integration	Promising localized delivery systems for CNS repair	Bae et al., 2021; Betancur et al., 2017; Kim et al., 2023; Zheng et al., 2021
Nanotechnology-assisted delivery	PLGA nanoparticles, nanodendriplexes, niosomal nanoparticles	Sustained release and targeted modulation of inflammatory pathways	Enhanced endogenous repair and graft efficacy	Facilitates precision delivery and cargo stabilization	Narouiepour et al., 2022; Mayilsamy et al., 2020; Zamproni et al., 2017
Electrospinning and 3D-printing platforms	Polydopamine-functionalized nanofibers, 3D-printed exosome implants	Sustained local release of therapeutic cargo	Promotion of neurovascular remodeling and structural regeneration	Strong potential for scalable tissue-engineered products	Zhang et al., 2024; Li et al., 2024; Liu et al., 2022
Cell or exosome preconditioning	Hypoxia-pretreated MSC-Exos, DHA supplementation, BDNF-overexpressing MSCs	Enhancement of therapeutic potency under hostile injury conditions	Improved neuroregeneration and functional recovery	Practical strategy for potency enhancement before transplantation	Liu et al., 2022; Ghazale et al., 2018; van Velthoven et al., 2013
Physical adjunctive therapies	Hyperbaric oxygen therapy, electrical stimulation	Optimization of metabolic and microenvironmental conditions	Enhanced stem cell migration, proliferation, and axonal remodeling	Attractive non-pharmacological combinatorial approach	Park et al., 2021; Yang et al., 2017; Zhou et al., 2016
Growth factor-based combination therapy	SCF + G-CSF, SCF + G-CSF + FLT3L, PEP-1-SOD1	Augmentation of endogenous neurorepair and anti-oxidative defense	Reduced neurodegeneration and enhanced remyelination	Particularly valuable for chronic and neonatal TBI	Jia et al., 2018; He et al., 2020; Qiu et al., 2023; Posod et al., 2019
Translational and clinical strategies	WJ-MSC trials, MSC-derived NSC-like cells, pediatric BMMNC therapy	Validation of feasibility and clinical safety	Functional improvement and neurotrophic factor elevation	Critical bridge from experimental therapy to clinical implementation	Saboori et al., 2024; Dewan et al., 2018; Kabatas et al., 2024, 2020; Wang et al., 2017

To maximize the utility of transplanted cells, combinatorial interventions focus on mitigating the harsh post-traumatic environment. Complex multimodal paradigms, such as the simultaneous use of embryonic NSC therapy, progesterone, and an enriched environment, further illustrate that addressing multiple pathological facets concurrently leads to superior cell survival and synaptic integration compared to monotherapies (Nudi et al., 2015). Ultimately, these advanced combined strategies aim to bridge the gap between experimental research and clinical application, emphasizing the need for standardized delivery and host environment optimization to ensure consistent therapeutic efficacy in diverse brain injury scenarios (Wagenaar et al., 2017).

In addition to manufacturing and characterization issues, the lack of reliable methods for tracking extracellular vesicles after administration remains a major challenge for clinical translation. Although fluorescent labeling, radionuclide imaging, and genetic labeling have been widely employed in experimental studies, each approach has inherent limitations in terms of labeling stability, specificity, and quantitative accuracy. As a result, the biodistribution, pharmacokinetics, cellular uptake, and long-term fate of administered vesicles have not yet been fully elucidated. Another critical issue is the absence of consistent reporting standards across preclinical studies. Greater adherence to the Minimal Information for Studies of Extracellular Vesicles (MISEV) guidelines would improve the consistency of experimental design, isolation procedures, vesicle characterization, and functional evaluation, thereby enhancing the reproducibility and comparability of published findings (Welsh et al., 2024). Establishing standardized methodologies will be essential for translating encouraging preclinical evidence into safe and effective clinical therapies for traumatic brain injury.

**Figure 3:**
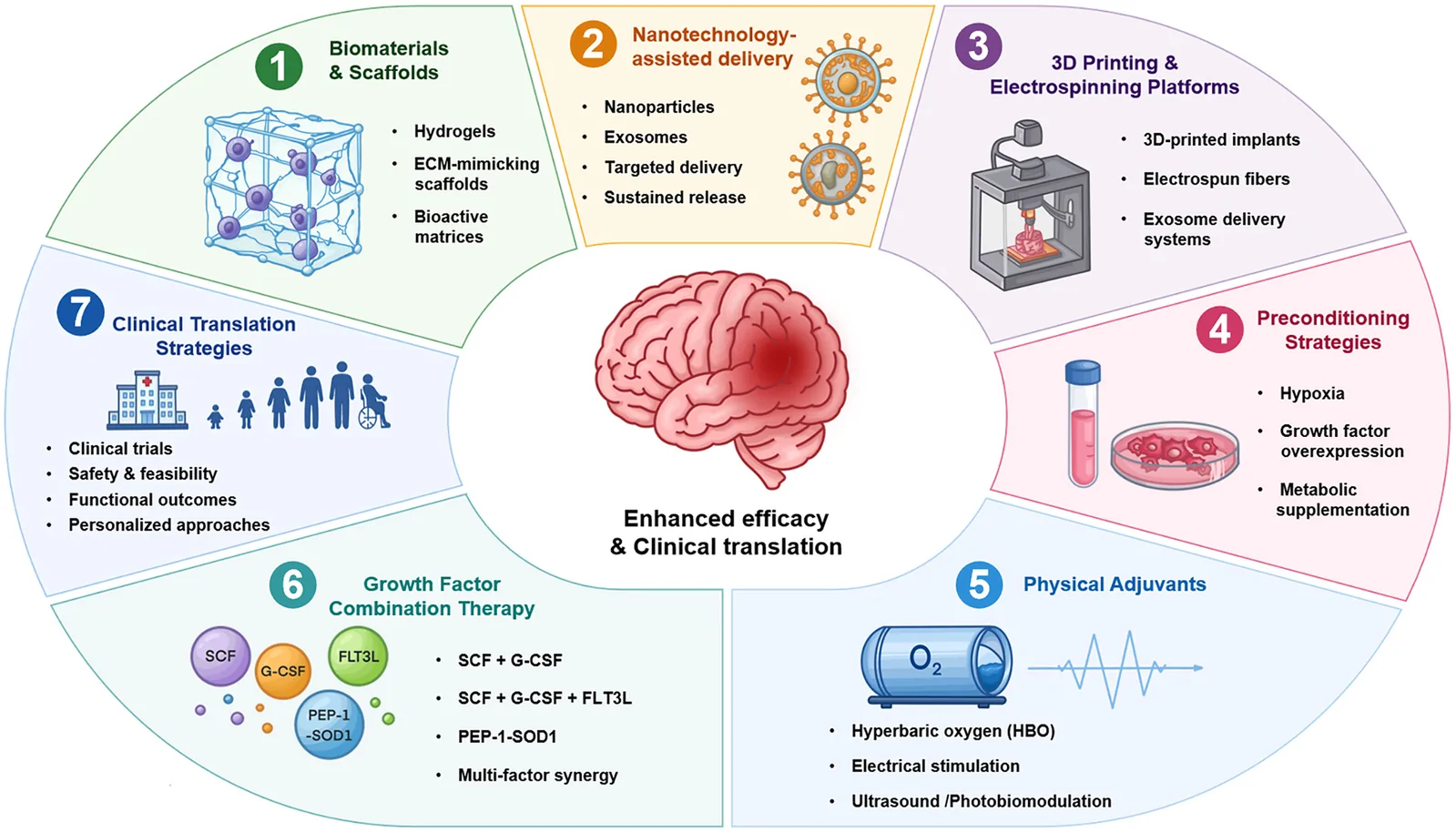
Engineering and combination strategies for enhancing stem cell therapy in traumatic brain injury.

Importantly, the efficacy of stem cell therapy can be further amplified by biomaterial engineering, nanoscale delivery, preconditioning, and multimodal adjunctive interventions. Figure 3 highlights the main strategies currently being explored to improve therapeutic potency and translational feasibility.

## 5 Translational and clinical perspectives

### 5.1 Clinical progress in stem cell therapy for TBI

The landscape of regenerative medicine for TBI remains heavily centered on stem cell-based interventions, with MSCs serving as a cornerstone for mitigating the secondary injury cascade. BM-MSCs demonstrate profound immunomodulatory and regenerative potential, which is essential for attenuating the prolonged neuroinflammatory response following TBI (Cozene et al., 2021). These multi-target effects allow MSC therapy to address various pathological manifestations, including the reduction of oxidative stress and the establishment of a neuroprotective environment through complex paracrine signaling (Zhang et al., 2022). Recent translational frameworks have established that transplanted stem cells operate primarily through these paracrine signaling networks, immunomodulatory actions, and neurotrophic support to preserve vulnerable neural networks rather than relying on direct cell replacement (Creel et al., 2026). Effective sequestration of TBI-induced inflammation is a critical mechanism through which these cells limit lesion expansion and modulate systemic immune activation to prevent further tissue degradation (Borlongan and Rosi, 2022). In addition to whole-cell transplantation, hUC-MSC-derived exosomes have emerged as potent mediators of neurological recovery, specifically by suppressing the pro-inflammatory activation of microglia and astrocytes within the injury site (Cui et al., 2022a). The clinical potential of MSC-EVs is further enhanced by their ability to traverse the BBB via intravenous delivery, facilitating the targeted transport of therapeutic miRNAs and growth factors to the damaged parenchyma (Xiong et al., 2024). Specifically, EVs derived from clonal MSCs have shown significant efficacy in preserving brain integrity by reducing cistauosis and neuronal apoptosis in weight-drop models of injury (Amini et al., 2023).

Complementing these findings, the stem cell secretome, including EVs and conditioned media, has proven effective in reducing neuroinflammation and mitigating structural damage in diverse preclinical settings (Muhammad et al., 2022). To circumvent invasive delivery limitations, the intranasal administration of ASC-CCM has been introduced to target disease-associated microglia (DAM) phenotypes. This secretome formulation effectively dampens chronic neuroinflammation by downregulating critical DAM markers such as APOE, TYROBP, and TREM2, which suppresses reactive astrogliosis, reduces apoptosis, and successfully rescues short- and long-term memory impairments (Rasiah et al., 2026).

State-of-the-art bioengineering strategies have further augmented therapeutic retention and tissue repair through genetic modifications and innovative biomaterials. For instance, the engineering of YTHDF1-modified NSCs represents a powerful genetic optimization frontier. Overexpression of the m6A reader YTHDF1 drastically enhances the proliferation and neuronal differentiation of transplanted NSCs via the PI3K/AKT pathway, protecting against oxidative stress and expediting hippocampal-dependent functional recovery (Zhang et al., 2026). Concurrently, structural biomaterial integration has resolved cell retention challenges by encapsulating therapeutic cells within a decellularized porcine dural extracellular matrix (ECM) hydrogel. This specific bioengineered matrix provides a supportive microenvironment that sustains NSC delivery, significantly curtails reactive gliosis, decreases GFAP expression, and yields prominent long-term motor improvements (Lee-Reeves et al., 2026). Expanding beyond lineage-committed cells, hiPSC-derived cortical organoids offer a revolutionary tissue-engineering modality to reconstruct physically disrupted cortical architecture. When grafted into the mechanically injured motor cortex, these hiPSC-derived cortical grafts selectively project to canonical efferent motor pathway targets and establish functional de novo synaptic connections characterized by the colocalization of post-synaptic density protein 95 (PSD95) with host neuronal cell bodies, which promotes full contralateral forelimb motor recovery (Gladen and Lybrand, 2026).

This foundational work has paved the way for human translation, as evidenced by a decade of clinical trials which collectively establish that stem cell therapies are safe and feasible for TBI patients (Saboori et al., 2024). Recent Phase I clinical data regarding WJ-MSCs further validate the safety of multi-route administration, reporting significant functional gains as measured by the Glasgow Coma Scale and other clinical metrics (Kabatas et al., 2024). These outcomes are exemplified by pilot studies where the targeted delivery of these cells successfully facilitated functional recovery in patients suffering from severe, persistent neurological deficits (Kabatas et al., 2020). Highlighting the most rigorous phase outcomes in current human prospective trials, evidence-mapped clinical registries confirm that the stereotactic intracerebral implantation of SB623 cells yields highly robust, methodologically sound signals for successfully reversing chronic motor deficits following TBI (Lepski and Arévalo, 2026).

Building directly upon these findings in TBI, the clinical translation of stem cell therapies has progressively expanded to evaluate therapeutic safety and efficacy across broader neurological conditions. Stereotactic intracranial implantation of SB623 cells demonstrates Class I evidence of safety and significant motor improvements, particularly at an optimal dose of 5.0 × 10^6^ cells (Kawabori et al., 2021). To evaluate these motor outcomes accurately, triangulated minimally clinically important differences have been established for the Disability Rating Scale and Fugl-Meyer subscales (McCrea et al., 2021). Furthermore, extended clinical evaluations confirm that cell therapy can safely modify long-term chronic neurological deficits well beyond 1 year post-injury (Okonkwo et al., 2024). Building on these insights from neurotrauma, ongoing investigations including phase 1 and 2 evaluations of allogeneic human dental pulp stem cells registered on clinicaltrials.gov continue to explore optimal dosing regimens and functional recovery endpoints for acute ischemic stroke (Suda et al., 2022). When evaluating systemic safety in other neurological conditions, studies demonstrate that intravenous mesenchymal stem cell infusion can induce transient D-dimer elevations without clinical thrombosis when managed with prophylactic anticoagulation (Hoang et al., 2024). Dose-escalation trials involving bone marrow-derived mesenchymal stem cells in acute intracerebral hemorrhage demonstrate favorable safety profiles and initial modulations of inflammatory cytokines (Durand et al., 2024). Direct comparative trials in ischemic stroke sequelae show that both intravenous and intrathecal umbilical cord-derived mesenchymal stem cell administrations significantly enhance neurological recovery, motor function, and quality of life (Nguyen et al., 2025). Additionally, early-phase dose-escalation trials further establish the safety and tolerability of intravenous umbilical cord-derived mesenchymal stem cell infusions up to 200 million cells in ischemic stroke (Cui et al., 2026).

### 5.2 Stem cell therapy in pediatric and developmental brain injury

Pediatric TBI and neonatal insults involve a complex interplay between injury mechanisms and an actively developing neural environment, which offers both unique vulnerabilities and a higher potential for neuroplasticity compared to the adult brain (Lengel et al., 2020). The administration of autologous BMMNCs in pediatric TBI cases has been established as a safe clinical approach, primarily functioning by attenuating the systemic inflammatory response and reducing pro-inflammatory cytokine production (Dewan et al., 2018). In the context of preterm brain injury, MSC-EVs provide a robust cell-free therapeutic alternative by effectively suppressing microglial activation and preserving long-term cognitive and behavioral functions after inflammation-induced damage (Drommelschmidt et al., 2017). Furthermore, the success of neonatal therapy is closely associated with the brain's endogenous capacity for repair, where specific biomarkers such as SCF and G-CSF mediate the mobilization and targeted migration of stem cells to hypoxic-ischemic lesions (Parry and Peeples, 2018). However, the pediatric population necessitates specialized protocols; the efficacy of stem cell interventions is highly dependent on the precise timing of the therapeutic window and weight-adjusted dosage, highlighting the need for age-specific safety assessments in clinical translation (Lin et al., 2023).

Furthermore, considering the distinct tissue plasticity and dynamic mechanical environment of the immature brain parenchyma, the design of bioengineered scaffolds for pediatric TBI requires careful optimization. Future translational strategies must address whether the Young's modulus of these delivery platforms should be dynamically adjusted or temporally tuned to accommodate the rapid development and compliance of juvenile brain tissues, thereby ensuring optimal cell survival and functional integration.

### 5.3 Lessons from related CNS injury models

The therapeutic principles established in other acute brain injury models provide a foundational mechanism for extrapolating stem cell efficacy to TBI. In ischemic environments, ASK1 serves as a pivotal molecular switch regulating NSC differentiation into neurons, suggesting that modulating stress-activated pathways is essential for functional integration (Song et al., 2013). The restorative potential of NSC transplantation is further evidenced by its ability to activate the Wnt signaling pathway, which significantly attenuates cerebral infarct expansion and reduces cellular apoptosis (Sun et al., 2025).

Beyond direct cell replacement, paracrine signaling via NSC-derived exosomes enriched with miR-150-3p has been shown to exert neuroprotective effects in hypoxic-ischemic injury by specifically targeting CASP2 to inhibit neuronal death (Luo et al., 2022). This mechanism of microRNA-mediated protection is also observed in subarachnoid hemorrhage, where MSC-EVs deliver miR-18a-5p to block the ENC1/p62 axis, thereby mitigating oxidative stress and endoplasmic reticulum stress (Zhang et al., 2023b).

Furthermore, mesenchymal stem cell interventions in neonatal hypoxic-ischemic models demonstrate a robust capacity to stimulate endogenous neurogenesis and the regeneration of oligodendrocytes (van Velthoven et al., 2010). The efficacy of these treatments is often augmented by specialized delivery or combination protocols; for instance, intranasal administration of MSCs overexpressing BDNF has been found to significantly reduce gray and white matter loss following neonatal stroke (van Velthoven et al., 2013). Finally, the synergistic application of UC-MSC transplantation and HBO therapy facilitates enhanced structural repair, illustrating that common mechanisms of neuroprotection and microenvironmental remodeling are shared across diverse CNS injuries (Zhou et al., 2016).

## 6 Current challenges and future directions

Despite the therapeutic potential of stem cell interventions for TBI, several critical hurdles limit their clinical translation. A primary challenge is the significant heterogeneity of cell sources, as substantial ambiguity remains regarding the selection of the most effective cell lineage, the determination of precise dosages, and the optimal therapeutic window for administration (Khazaal et al., 2025). A major technical bottleneck is the exceptionally low survival and retention rate of transplanted cells within the injury niche. The hostile post-TBI microenvironment, often saturated with pro-inflammatory cytokines and oxidative stress, typically leads to the premature death of grafted cells before they can achieve functional integration (Zhou et al., 2019). Furthermore, delivery routes remain a point of contention, as systemic administration frequently fails to ensure adequate cell homing to the damaged brain parenchyma, resulting in inconsistent clinical outcomes (Saboori et al., 2024). Evaluation standards also lack uniformity; conventional cellular therapies are often hindered by low throughput and concerns over long-term safety, such as potential tumorigenicity or ethical barriers associated with specific cell origins (Cui et al., 2022b). Finally, the complex interaction between the host environment and transplanted cells, including how neurological states like posttraumatic hyperexcitability influence cellular behavior, necessitates more rigorous longitudinal monitoring to confirm genuine functional restoration (Beretta et al., 2017).

The precise therapeutic mechanism of stem cell interventions remains a subject of ongoing debate, specifically regarding the balance between direct cell replacement and paracrine signaling. While early research emphasized the necessity of lineage differentiation to replace lost neurons, the low rate of long-term engraftment suggests that “bystander” effects may be the primary driver of recovery (Reis et al., 2017). Transcriptomic analysis has revealed that hMSC treatment works by normalizing cortical gene expression, particularly within pathways related to immune cell recruitment and signaling (Darkazalli et al., 2017).

Despite the therapeutic promise of stem cell and EVs, comprehensive safety evaluations remain crucial to address potential adverse events (Thompson et al., 2020). Systemic administration of cellular therapies frequently induces transient fevers as a primary side effect, yet it does not significantly escalate the incidence of acute infusional toxicities, severe infections, or long-term malignancies (Petrus-Reurer et al., 2021). Immune-mediated reactions and hypersensitivity responses can occasionally occur following allogeneic transplantation, necessitating stringent donor-recipient matching and purity assessments (Kalluri, 2024). Furthermore, intravascular delivery carries inherent biophysical hazards, including procoagulant effects and microvascular embolism risks, which heavily depend on cell dosage, infusion speed, and aggregation status (Takakura et al., 2024). Dose-dependent toxicity profiles underscore the necessity of establishing rigorous therapeutic windows, as excessive concentrations can trigger localized ischemia or systemic inflammatory cascades (Leonov et al., 2025). Finally, although direct evidence of neoplastic transformation remains strictly controlled, the inherent proliferative capacity of stem cell populations mandates long-term surveillance for potential carcinogenic effects prior to widespread clinical adoption (Xu et al., 2025).

Future research must prioritize the transition from experimental proof-of-concept to standardized clinical application. Systematic meta-analyses of the stem cell secretome highlight the urgent need for uniform bioprocessing protocols and precise delivery methods to minimize the variability seen in preclinical structural and functional outcomes (Muhammad et al., 2022). Clinical safety has been established for autologous MSC-derived NSC-like cells, yet the correlation between neurological recovery and the sustained elevation of neurotrophic factors such as NGF and BDNF requires further longitudinal validation (Wang et al., 2017). Finally, phase I results using WJ-MSCs underscore that future trials must focus on the standardization of cell preparation and the design of rigorous clinical endpoints to ensure the long-term efficacy and safety of these regenerative strategies (Kabatas et al., 2024).

## 7 Conclusion

Stem cell-based therapies are reshaping the therapeutic landscape of TBI by offering regenerative and microenvironment-modulating strategies that extend beyond conventional symptomatic management. Accumulating evidence indicates that MSCs, NSCs, and cell-free derivatives such as EVs can simultaneously target multiple pathological processes, including neuroinflammation, neuronal loss, vascular dysfunction, and impaired neural plasticity. Recent advances in biomaterials, nanotechnology, and engineered delivery systems have further expanded the therapeutic potential and translational feasibility of these approaches.

Despite substantial progress, important challenges remain before routine clinical application can be achieved. Issues related to cell heterogeneity, therapeutic standardization, delivery optimization, long-term safety, and patient stratification continue to limit translational success. Future development will require closer integration of mechanistic understanding, bioengineering innovation, and rigorously designed clinical studies. With continued advances in regenerative medicine and precision therapeutics, stem cell-based interventions may ultimately provide a viable strategy for durable neurological repair after TBI.

## References

[B1] Alam A. Singh T. Kayhanian S. Tjerkaski J. Garcia N. M. Carpenter K. L. H. . (2023). Modeling the inflammatory response of traumatic brain injury using human induced pluripotent stem cell derived microglia. J. Neurotrauma 40, 2164–2173. doi: 10.1089/neu.2022.0508 37261979

[B2] Amini A. Shekari F. Assar Kashani S. Eslami N. Nazari A. Tofigh N. . (2023). Clonal mesenchymal stem cell-derived extracellular vesicles improve mouse model of weight drop-induced traumatic brain injury through reducing cistauosis and apoptosis. Exp. Neurol. 367, 114467. doi: 10.1016/j.expneurol.2023.114467 37302747

[B3] Bae M. Hwang D. W. Ko M. K. Jin Y. Shin W. J. Park W. . (2021). Neural stem cell delivery using brain-derived tissue-specific bioink for recovering from traumatic brain injury. Biofabrication 13:10.1088/1758-5090/ac293f. doi: 10.1088/1758-5090/ac293f 34551404

[B4] Bayat Tork M. A. Saberifar M. Joneidi Yekta H. Hajinejad M. Hosseini Ravandi H. Gorji A. . (2025). Nano-scaffold containing functional motif of stromal cell-derived factor 1 enhances neural stem cell behavior and synaptogenesis in traumatic brain injury. Sci. Rep. 15:5811. doi: 10.1038/s41598-025-85698-5 39962142 PMC11832925

[B5] Beretta S. Cunningham K. M. Haus D. L. Gold E. M. Perez H. Lopez-Velazquez L. . (2017). Effects of human ES-derived neural stem cell transplantation and kindling in a rat model of traumatic brain injury. Cell Transplant. 26, 1247–1261. doi: 10.1177/0963689717714107 28933218 PMC5657732

[B6] Betancur M. I. Mason H. D. Alvarado-Velez M. Holmes P. V. Bellamkonda R. V. Karumbaiah L. . (2017). Chondroitin sulfate glycosaminoglycan matrices promote neural stem cell maintenance and neuroprotection post-traumatic brain injury. ACS Biomater. Sci. Eng. 3, 420–430. doi: 10.1021/acsbiomaterials.6b00805 29744379 PMC5937277

[B7] Bonsack B. Corey S. Shear A. Heyck M. Cozene B. Sadanandan N. . (2020). Mesenchymal stem cell therapy alleviates the neuroinflammation associated with acquired brain injury. CNS Neurosci. Ther. 26, 603–615. doi: 10.1111/cns.13378 32356605 PMC7248547

[B8] Borlongan M. C. Rosi S. (2022). Stem Cell Therapy for Sequestration of traumatic brain injury-induced inflammation. Int. J. Mol. Sci. 23:10286. doi: 10.3390/ijms231810286 36142198 PMC9499317

[B9] Chang J. Phelan M. Cummings B. J. (2015). A meta-analysis of efficacy in pre-clinical human stem cell therapies for traumatic brain injury. Exp. Neurol. 273, 225–233. doi: 10.1016/j.expneurol.2015.08.020 26342754

[B10] Chen K. H. Shao P. L. Li Y. C. Chiang J. Y. Sung P. H. Chien H. W. . (2020). Human umbilical cord-derived mesenchymal stem cell therapy effectively protected the brain architecture and neurological function in rat after acute traumatic brain injury. Cell Transplant. 29:963689720929313. doi: 10.1177/0963689720929313 33169616 PMC7784577

[B11] Cheng S. Lu Q. Liu Q. Ma Y. Chen J. Lu D. . (2024). Synergistic effects of repeated transcranial magnetic stimulation and mesenchymal stem cells transplantation on alleviating neuroinflammation and PANoptosis in cerebral ischemia. J. Neuroinflammation 21:311. doi: 10.1186/s12974-024-03302-5 39616364 PMC11607903

[B12] Cozene B. Sadanandan N. Farooq J. Kingsbury C. Park Y. J. Wang Z. J. . (2021). Mesenchymal stem cell-induced anti-neuroinflammation against traumatic brain injury. Cell Transplant. 30:9636897211035715. doi: 10.1177/09636897211035715 34559583 PMC8485159

[B13] Creel W. T. Randhawa S. Hartman R. E. (2026). Stem cell therapy for traumatic brain injury: translational mechanisms, biomarkers, and clinical trials. Stem Cell Rev. Rep. 22, 2808–2823. doi: 10.1007/s12015-026-11175-9 42313238 PMC13354699

[B14] Cui L. Luo W. Jiang W. Li H. Xu J. Liu X. . (2022a). Human umbilical cord mesenchymal stem cell-derived exosomes promote neurological function recovery in rat after traumatic brain injury by inhibiting the activation of microglia and astrocyte. Regen. Ther. 21, 282–287. doi: 10.1016/j.reth.2022.07.005 36092501 PMC9440059

[B15] Cui L. Saeed Y. Li H. Yang J. (2022b). Regenerative medicine and traumatic brain injury: from stem cell to cell-free therapeutic strategies. Regen. Med. 17, 37–53. doi: 10.2217/rme-2021-0069 34905963

[B16] Cui L. Xiao M. Zhang Q. Liu H. Ren Y. Ma G. . (2026). Intravenous human umbilical cord-derived mesenchymal stem cell transplantation for ischemic stroke (MERIT): a phase 1 trial. Cell Rep. Med. 7:102836. doi: 10.1016/j.xcrm.2026.102836 42208543 PMC13293954

[B17] Dabrowska S. Andrzejewska A. Strzemecki D. Muraca M. Janowski M. Lukomska B. . (2019). Human bone marrow mesenchymal stem cell-derived extracellular vesicles attenuate neuroinflammation evoked by focal brain injury in rats. J. Neuroinflammation 16, 216. doi: 10.1186/s12974-019-1602-5 31722731 PMC6852925

[B18] Dai Y. Sun F. Zhu H. Liu Q. Xu X. Gong P. . (2019). Effects and mechanism of action of neonatal versus adult astrocytes on neural stem cell proliferation after traumatic brain injury. Stem Cells 37, 1344–1356. doi: 10.1002/stem.3060 31287930

[B19] Darkazalli A. Vied C. Badger C. D. Levenson C. W. (2017). Human mesenchymal stem cell treatment normalizes cortical gene expression after traumatic brain injury. J. Neurotrauma 34, 204–212. doi: 10.1089/neu.2015.4322 27161121

[B20] Das M. Mayilsamy K. Mohapatra S. S. Mohapatra S. (2019). Mesenchymal stem cell therapy for the treatment of traumatic brain injury: progress and prospects. Rev. Neurosci. 30, 839–855. doi: 10.1515/revneuro-2019-0002 31203262

[B21] Deng T. Ding R. Wang Y. Chen Y. Sun H. Zheng M. . (2024). Mapping knowledge of the stem cell in traumatic brain injury: a bibliometric and visualized analysis. Front. Neurol. 15:1301277. doi: 10.3389/fneur.2024.1301277 38523616 PMC10957745

[B22] Dewan S. Schimmel S. Borlongan C. V. (2018). Treating childhood traumatic brain injury with autologous stem cell therapy. Expert Opin. Biol. Ther. 18, 515–524. doi: 10.1080/14712598.2018.1439473 29421958 PMC6086119

[B23] Drommelschmidt K. Serdar M. Bendix I. Herz J. Bertling F. Prager S. . (2017). Mesenchymal stem cell-derived extracellular vesicles ameliorate inflammation-induced preterm brain injury. Brain Behav. Immun. 60, 220–232. doi: 10.1016/j.bbi.2016.11.011 27847282

[B24] Durand N. C. Kim H. G. Patel V. N. Turnbull M. T. Siegel J. L. Hodge D. O. . (2024). Mesenchymal stem cell therapy in acute intracerebral hemorrhage: a dose-escalation safety and tolerability trial. Neurocrit. Care 41, 59–69. doi: 10.1007/s12028-023-01897-w 38114796 PMC11335835

[B25] Furmanski O. Nieves M. D. Doughty M. L. (2019). Controlled cortical impact model of mouse brain injury with therapeutic transplantation of human induced pluripotent stem cell-derived neural cells. J. Vis. Exp. 10.3791/59561. doi: 10.3791/59561 31355806

[B26] Ghazale H. Ramadan N. Mantash S. Zibara K. El-Sitt S. Darwish H. . (2018). Docosahexaenoic acid (DHA) enhances the therapeutic potential of neonatal neural stem cell transplantation post-traumatic brain injury. Behav. Brain Res. 340, 1–13. doi: 10.1016/j.bbr.2017.11.007 29126932

[B27] Gladen M. D. Lybrand Z. R. (2026). Human induced pluripotent stem cell–derived cortical grafts rebuild motor circuits after brain injury. Neural Regen. Res. 10.4103/NRR.NRR-D-25-01272. doi: 10.4103/NRR.NRR-D-25-01272 42199138

[B28] Harting M. T. Jimenez F. Xue H. Fischer U. M. Baumgartner J. Dash P. K. . (2009). Intravenous mesenchymal stem cell therapy for traumatic brain injury. J. Neurosurg. 110, 1189–1197. doi: 10.3171/2008.9.JNS08158 19301973 PMC2889620

[B29] He J. Russell T. Qiu X. Hao F. Kyle M. Chin L. . (2020). The contribution of stem cell factor and granulocyte colony-stimulating factor in reducing neurodegeneration and promoting neurostructure network reorganization after traumatic brain injury. Brain Res. 1746:147000. doi: 10.1016/j.brainres.2020.147000 32579949

[B30] Heile A. Brinker T. (2011). Clinical translation of stem cell therapy in traumatic brain injury: the potential of encapsulated mesenchymal cell biodelivery of glucagon-like peptide-1. Dialogues Clin. Neurosci. 13, 279–286. doi: 10.31887/DCNS.2011.13.2/aheile 22034462 PMC3182013

[B31] Herz J. Koster C. Reinboth B. S. Dzietko M. Hansen W. Sabir H. . (2018). Interaction between hypothermia and delayed mesenchymal stem cell therapy in neonatal hypoxic-ischemic brain injury. Brain Behav. Immun. 70, 118–130. doi: 10.1016/j.bbi.2018.02.006 29454023

[B32] Hoang V. T. Le D. S. Hoang D. M. Phan T. T. K. Ngo L. A. T. Nguyen T. K. . (2024). Impact of tissue factor expression and administration routes on thrombosis development induced by mesenchymal stem/stromal cell infusions: re-evaluating the dogma. Stem Cell Res. Ther. 15:56. doi: 10.1186/s13287-023-03582-3 38414067 PMC10900728

[B33] Hu Z. Gajavelli S. Spurlock M. S. Mahavadi A. Quesada L. S. Gajavelli G. R. . (2020). Human neural stem cell transplant location-dependent neuroprotection and motor deficit amelioration in rats with penetrating traumatic brain injury. J. Trauma Acute Care Surg. 88, 477–485. doi: 10.1097/TA.0000000000002510 31626023 PMC7098436

[B34] Jha K. A. Gentry J. Del Mar N. A. Reiner A. Sohl N. Gangaraju R. . (2021). Adipose tissue-derived mesenchymal stem cell concentrated conditioned medium alters the expression pattern of glutamate regulatory proteins and aquaporin-4 in the retina after mild traumatic brain injury. J. Neurotrauma 38, 1702–1716. doi: 10.1089/neu.2020.7309 33183134 PMC8165480

[B35] Jia J. Chen F. Wu Y. (2018). Recombinant PEP-1-SOD1 improves functional recovery after neural stem cell transplantation in rats with traumatic brain injury. Exp. Ther. Med. 15, 2929–2935. doi: 10.3892/etm.2018.5781 29599832 PMC5867477

[B36] Kabatas S. Civelek E. Boyali O. Sezen G. B. Ozdemir O. Bahar-Ozdemir Y. . (2024). Safety and efficiency of Wharton's Jelly-derived mesenchymal stem cell administration in patients with traumatic brain injury: first results of a phase I study. World J. Stem Cells 16, 641–655. doi: 10.4252/wjsc.v16.i6.641 38948099 PMC11212551

[B37] Kabatas S. Civelek E. Sezen G. B. Kaplan N. Savrunlu E. C. Cetin E. . (2020). Functional recovery after wharton's jelly-derived mesenchymal stem cell administration in a patient with traumatic brain injury: a pilot study. Turk. Neurosurg. 30, 914–922. doi: 10.5137/1019-5149.JTN.31732-20.1 33216342

[B38] Kalluri R. (2024). The biology and function of extracellular vesicles in immune response and immunity. Immunity 57, 1752–1768. doi: 10.1016/j.immuni.2024.07.009 39142276 PMC11401063

[B39] Kang E. M. Jia Y. B. Wang J. Y. Wang G. Y. Chen H. J. Chen X. Y. . (2022). Downregulation of microRNA-124-3p promotes subventricular zone neural stem cell activation by enhancing the function of BDNF downstream pathways after traumatic brain injury in adult rats. CNS Neurosci. Ther. 28, 1081–1092. doi: 10.1111/cns.13845 35481944 PMC9160452

[B40] Kappy N. S. Chang S. Harris W. M. Plastini M. Ortiz T. Zhang P. . (2018). Human adipose-derived stem cell treatment modulates cellular protection in both in vitro and in vivo traumatic brain injury models. J. Trauma Acute Care Surg. 84, 745–751. doi: 10.1097/TA.0000000000001770 29251705

[B41] Kawabori M. Weintraub A. H. Imai H. Zinkevych I. McAllister P. Steinberg G. K. . (2021). Cell therapy for chronic TBI: interim analysis of the randomized controlled STEMTRA trial. Neurology 96, e1202–e1214. doi: 10.1212/WNL.0000000000011450 33397772 PMC8055341

[B42] Khazaal A. Q. Ismaeel H. M. Cheah P. S. Nordin N. (2025). Cellular stem cell therapy for treating traumatic brain injury: strategies for enhancement of therapeutic efficacy. Mol. Neurobiol. 62, 8359–8380. doi: 10.1007/s12035-025-04778-9 40000574

[B43] Kim J. T. Cho S. M. Youn D. H. Hong E. P. Park C. H. Lee Y. . (2023). Therapeutic effect of a hydrogel-based neural stem cell delivery sheet for mild traumatic brain injury. Acta Biomater. 167, 335–347. doi: 10.1016/j.actbio.2023.06.027 37356785

[B44] Kim J. T. Kim T. Y. Youn D. H. Han S. W. Park C. H. Lee Y. . (2022). Human embryonic stem cell-derived cerebral organoids for treatment of mild traumatic brain injury in a mouse model. Biochem. Biophys. Res. Commun. 635, 169–178. doi: 10.1016/j.bbrc.2022.10.045 36274367

[B45] Kodali M. Madhu L. N. Reger R. L. Milutinovic B. Upadhya R. Attaluri S. . (2023). A single intranasal dose of human mesenchymal stem cell-derived extracellular vesicles after traumatic brain injury eases neurogenesis decline, synapse loss, and BDNF-ERK-CREB signaling. Front. Mol. Neurosci. 16:1185883. doi: 10.3389/fnmol.2023.1185883 37284464 PMC10239975

[B46] Lan Z. Tan F. He J. Liu J. Lu M. Hu Z. . (2024). Curcumin-primed olfactory mucosa-derived mesenchymal stem cells mitigate cerebral ischemia/reperfusion injury-induced neuronal PANoptosis by modulating microglial polarization. Phytomedicine 129:155635. doi: 10.1016/j.phymed.2024.155635 38701541

[B47] Lee J. Y. Acosta S. Tuazon J. P. Xu K. Nguyen H. Lippert T. . (2019). Human parthenogenetic neural stem cell grafts promote multiple regenerative processes in a traumatic brain injury model. Theranostics 9, 1029–1046. doi: 10.7150/thno.29868 30867814 PMC6401413

[B48] Lee-Reeves C. Gregory H. N. Gorup D. Chan M. Roche R. Irfan M. . (2026). A dural extracellular matrix hydrogel with neural stem cells improves recovery from traumatic brain injury in mice. ACS Biomater. Sci. Eng. 10.1021/acsbiomaterials.6c00319. doi: 10.1021/acsbiomaterials.6c00319 42391517

[B49] Lengel D. Sevilla C. Romm Z. L. Huh J. W. Raghupathi R. (2020). Stem cell therapy for pediatric traumatic brain injury. Front. Neurol. 11:601286. doi: 10.3389/fneur.2020.601286 33343501 PMC7738475

[B50] Leonov G. E. Grinchevskaya L. R. Makhnach O. V. Samburova M. V. Goldshtein D. V. Salikhova D. I. . (2025). Safety assessment of stem cell-based therapies: current standards and advancing frameworks. Cells 14:1660. doi: 10.3390/cells14211660 41227306 PMC12609812

[B51] Lepski G. Arévalo A. (2026). Mesenchymal stromal/stem cells for neurological disorders in humans: an evidence-mapped clinical review. Front. Cell. Neurosci. 20:1844360. doi: 10.3389/fncel.2026.1844360 42358489 PMC13290518

[B52] Li J. Li X. Li X. Liang Z. Wang Z. Shahzad K. A. . (2024). Local delivery of dual stem cell-derived exosomes using an electrospun nanofibrous platform for the treatment of traumatic brain injury. ACS Appl. Mater. Interfaces 16, 37497–37512. doi: 10.1021/acsami.4c05004 38980910

[B53] Lin W. Y. Wu K. H. Chen C. Y. Guo B. C. Chang Y. J. Lee T. A. . (2023). Stem cell therapy in children with traumatic brain injury. Int. J. Mol. Sci. 24:14706. doi: 10.3390/ijms241914706 37834152 PMC10573043

[B54] Ling Y. Ramalingam M. Lv X. Niu D. Zeng Y. Qiu Y. . (2024). Human neural stem cell secretome relieves endoplasmic reticulum stress-induced apoptosis and improves neuronal functions after traumatic brain injury in a rat model. J. Mol. Histol. 55, 329–348. doi: 10.1007/s10735-024-10192-7 38609527

[B55] Liu X. Wang J. Wang P. Zhong L. Wang S. Feng Q. . (2022). Hypoxia-pretreated mesenchymal stem cell-derived exosomes-loaded low-temperature extrusion 3D-printed implants for neural regeneration after traumatic brain injury in canines. Front. Bioeng. Biotechnol. 10:1025138. doi: 10.3389/fbioe.2022.1025138 36246376 PMC9562040

[B56] Luo H. Ye G. Liu Y. Huang D. Luo Q. Chen W. . (2022). miR-150-3p enhances neuroprotective effects of neural stem cell exosomes after hypoxic-ischemic brain injury by targeting CASP2. Neurosci. Lett. 779:136635. doi: 10.1016/j.neulet.2022.136635 35436510

[B57] Mayilsamy K. Markoutsa E. Das M. Chopade P. Puro D. Kumar A. . (2020). Treatment with shCCL20-CCR6 nanodendriplexes and human mesenchymal stem cell therapy improves pathology in mice with repeated traumatic brain injury. Nanomedicine 29:102247. doi: 10.1016/j.nano.2020.102247 32599163

[B58] McCrea M. A. Cramer S. C. Okonkwo D. O. Mattke S. Paadre S. Bates D. . (2021). Determining minimally clinically important differences for outcome measures in patients with chronic motor deficits secondary to traumatic brain injury. Expert Rev. Neurother. 21, 1051–1058. doi: 10.1080/14737175.2021.1968299 34402352

[B59] Muhammad S. A. Abbas A. Y. Imam M. U. Saidu Y. Bilbis L. S. (2022). Efficacy of stem cell secretome in the treatment of traumatic brain injury: a systematic review and meta-analysis of preclinical studies. Mol. Neurobiol. 59, 2894–2909. doi: 10.1007/s12035-022-02759-w 35230664

[B60] Narouiepour A. Ebrahimzadeh-Bideskan A. Rajabzadeh G. Gorji A. Negah S. S. (2022). Neural stem cell therapy in conjunction with curcumin loaded in niosomal nanoparticles enhanced recovery from traumatic brain injury. Sci. Rep. 12:3572. doi: 10.1038/s41598-022-07367-1 35246564 PMC8897489

[B61] Nguyen L. T. Nguyen T. T. N. Nguyen K. T. Phung L. N. Hoang V. T. Phan T. T. K. . (2025). Intrathecal versus intravenous umbilical cord mesenchymal stem cells for ischemic stroke sequelae. Stem Cells Transl. Med. 14:szaf063. doi: 10.1093/stcltm/szaf063 41277536 PMC12641229

[B62] Nudi E. T. Jacqmain J. Dubbs K. Geeck K. Salois G. Searles M. A. . (2015). Combining enriched environment, progesterone, and embryonic neural stem cell therapy improves recovery after brain injury. J. Neurotrauma 32, 1117–1129. doi: 10.1089/neu.2014.3618 25268854

[B63] Okonkwo D. O. McAllister P. Achrol A. S. Karasawa Y. Kawabori M. Cramer S. C. . (2024). Mesenchymal stromal cell implants for chronic motor deficits after traumatic brain injury: *post hoc* analysis of a randomized trial. Neurology 103:e209797. doi: 10.1212/WNL.0000000000209797 39231380 PMC11373674

[B64] Pang A. L. Xiong L. L. Xia Q. J. Liu F. Wang Y. C. Liu F. . (2017). Neural stem cell transplantation is associated with inhibition of apoptosis, Bcl-xL upregulation, and recovery of neurological function in a rat model of traumatic brain injury. Cell Transplant. 26, 1262–1275. doi: 10.1177/0963689717715168 28933221 PMC5657736

[B65] Park E. Lyon J. G. Alvarado-Velez M. Betancur M. I. Mokarram N. Shin J. H. . (2021). Enriching neural stem cell and anti-inflammatory glial phenotypes with electrical stimulation after traumatic brain injury in male rats. J. Neurosci. Res. 99, 1864–1884. doi: 10.1002/jnr.24834 33772860 PMC8360147

[B66] Parry S. M. Peeples E. S. (2018). The impact of hypoxic-ischemic brain injury on stem cell mobilization, migration, adhesion, and proliferation. Neural Regen. Res. 13, 1125–1135. doi: 10.4103/1673-5374.235012 30028311 PMC6065219

[B67] Petrus-Reurer S. Romano M. Howlett S. Jones J. L. Lombardi G. Saeb-Parsy K. . (2021). Immunological considerations and challenges for regenerative cellular therapies. Commun. Biol. 4:798. doi: 10.1038/s42003-021-02237-4 34172826 PMC8233383

[B68] Posod A. Wegleiter K. Neubauer V. Urbanek M. Huber E. Kiechl-Kohlendorfer U. . (2019). Short-, mid-, and long-term effect of granulocyte colony-stimulating factor/stem cell factor and Fms-related tyrosine kinase 3 ligand evaluated in an in vivo model of hypoxic-hyperoxic ischemic neonatal brain injury. Biomed Res. Int. 2019:5935279. doi: 10.1155/2019/5935279 31001556 PMC6436372

[B69] Qiu X. Ping S. Kyle M. Chin L. Zhao L. R. (2023). Stem cell factor and granulocyte colony-stimulating factor promote remyelination in the chronic phase of severe traumatic brain injury. Cells 12:705. doi: 10.3390/cells12050705 36899841 PMC10000780

[B70] Rasiah P. K. Ismael S. Elshaer S. Awad A. M. Salman M. Wang Z. . (2026). Mesenchymal stem cell secretome attenuates disease-associated microglial activation and cognitive decline in TBI-associated neuroinflammation. Neurochem. Int. 198:106210. doi: 10.1016/j.neuint.2026.106210 42342036

[B71] Reis C. Gospodarev V. Reis H. Wilkinson M. Gaio J. Araujo C. . (2017). Traumatic brain injury and stem cell: pathophysiology and update on recent treatment modalities. Stem Cells Int. 2017:6392592. doi: 10.1155/2017/6392592 28852409 PMC5568618

[B72] Saboori M. Riazi A. Taji M. Yadegarfar G. (2024). Traumatic brain injury and stem cell treatments: a review of recent 10 years clinical trials. Clin. Neurol. Neurosurg. 239:108219. doi: 10.1016/j.clineuro.2024.108219 38471197

[B73] Schantz S. L. Sneed S. E. Fagan M. M. Golan M. E. Cheek S. R. Kinder H. A. . (2024). Human-induced pluripotent stem cell-derived neural stem cell therapy limits tissue damage and promotes tissue regeneration and functional recovery in a pediatric piglet traumatic-brain-injury model. Biomedicines 12:1663. doi: 10.3390/biomedicines12081663 39200128 PMC11351842

[B74] Shin H. E. Lee W. J. Park K. S. Yu Y. Kim G. Roh E. J. . (2024). Repeated intrathecal injections of peripheral nerve-derived stem cell spheroids improve outcomes in a rat model of traumatic brain injury. Stem Cell Res. Ther. 15:314. doi: 10.1186/s13287-024-03874-2 39300591 PMC11414269

[B75] Song J. Cho K. J. Cheon S. Y. Kim S. - H. . (2013). Apoptosis signal-regulating kinase 1 (ASK1) is linked to neural stem cell differentiation after ischemic brain injury. Exp. Mol. Med. 45, e69–e69. doi: 10.1038/emm.2013.134 24357833 PMC3880461

[B76] Suda S. Nito C. Ihara M. Iguchi Y. Urabe T. Matsumaru Y. . (2022). Randomised placebo-controlled multicentre trial to evaluate the efficacy and safety of JTR-161, allogeneic human dental pulp stem cells, in patients with Acute Ischaemic stRoke (J-REPAIR). BMJ Open 12:e054269. doi: 10.1136/bmjopen-2021-054269 PMC9125710 35613802

[B77] Sun L. Lee J. Fine H. A. (2004). Neuronally expressed stem cell factor induces neural stem cell migration to areas of brain injury. J. Clin. Invest. 113, 1364–1374. doi: 10.1172/JCI200420001 15124028 PMC398428

[B78] Sun Y. Shen B. Wang T. Zhang G. Wang P. Huang H. . (2025). Restorative effects and mechanisms of neural stem cell transplantation on ischemic brain injury based on the Wnt signaling pathway. Int. J. Neurosci. 135, 219–227. doi: 10.1080/00207454.2023.2294261 38108312

[B79] Takakura Y. Hanayama R. Akiyoshi K. Futaki S. Hida K. Ichiki T. . (2024). Quality and safety considerations for therapeutic products based on extracellular vesicles. Pharm. Res. 41, 1573–1594. doi: 10.1007/s11095-024-03757-4 39112776 PMC11362369

[B80] Tang L. Xu Y. Wang L. Pan J. (2023). Adipose-derived stem cell exosomes ameliorate traumatic brain injury through the NLRP3 signaling pathway. Neuroreport 34, 677–684. doi: 10.1097/WNR.0000000000001941 37506308 PMC10399942

[B81] Thelin E. P. Hall C. E. Gupta K. Carpenter K. L. H. Chandran S. Hutchinson P. J. . (2018). Elucidating pro-inflammatory cytokine responses after traumatic brain injury in a human stem cell model. J. Neurotrauma 35, 341–352. doi: 10.1089/neu.2017.5155 28978285 PMC5784793

[B82] Thompson M. Mei S. H. J. Wolfe D. Champagne J. Fergusson D. Stewart D. J. . (2020). Cell therapy with intravascular administration of mesenchymal stromal cells continues to appear safe: an updated systematic review and meta-analysis. EClinicalMedicine 19, 100249. doi: 10.1016/j.eclinm.2019.100249 31989101 PMC6970160

[B83] van Velthoven C. T. Kavelaars A. van Bel F. Heijnen C. J. (2010). Mesenchymal stem cell treatment after neonatal hypoxic-ischemic brain injury improves behavioral outcome and induces neuronal and oligodendrocyte regeneration. Brain Behav. Immun. 24, 387–393. doi: 10.1016/j.bbi.2009.10.017 19883750

[B84] van Velthoven C. T. Sheldon R. A. Kavelaars A. Derugin N. Vexler Z. S. Willemen H. L. . (2013). Mesenchymal stem cell transplantation attenuates brain injury after neonatal stroke. Stroke 44, 1426–1432. doi: 10.1161/STROKEAHA.111.000326 23539530 PMC3694433

[B85] Wagenaar N. Nijboer C. H. van Bel F. (2017). Repair of neonatal brain injury: bringing stem cell-based therapy into clinical practice. Dev. Med. Child Neurol. 59, 997–1003. doi: 10.1111/dmcn.13528 28786482

[B86] Wang G. Wu H. L. Liu Y. P. Yan D. Q. Yuan Z. L. Chen L. . (2022). Pre-clinical study of human umbilical cord mesenchymal stem cell transplantation for the treatment of traumatic brain injury: safety evaluation from immunogenic and oncogenic perspectives. Neural Regen. Res. 17, 354–361. doi: 10.4103/1673-5374.317985 34269210 PMC8463980

[B87] Wang X. Seekaew P. Gao X. Chen J. (2016). Traumatic brain injury stimulates neural Stem cell proliferation via mammalian target of rapamycin signaling pathway activation. eNeuro 3:ENEURO.0162-16.2016. doi: 10.1523/ENEURO.0162-16.2016 PMC5089538 27822507

[B88] Wang Z. Luo Y. Chen L. Liang W. (2017). Safety of neural stem cell transplantation in patients with severe traumatic brain injury. Exp. Ther. Med. 13, 3613–3618. doi: 10.3892/etm.2017.4423 28588689 PMC5450816

[B89] Welsh J. A. Goberdhan D. C. I. O'Driscoll L. Buzas E. I. Blenkiron C. Bussolati B. . (2024). Minimal information for studies of extracellular vesicles (MISEV2023): From basic to advanced approaches. J. Extracell. Vesicles 13:e12404. doi: 10.1002/jev2.12404 38326288 PMC10850029

[B90] Williams A. M. Dennahy I. S. Bhatti U. F. Halaweish I. Xiong Y. Chang P. . (2019). Mesenchymal stem cell-derived exosomes provide neuroprotection and improve long-term neurologic outcomes in a swine model of traumatic brain injury and hemorrhagic shock. J. Neurotrauma 36, 54–60. doi: 10.1089/neu.2018.5711 29690826

[B91] Xiong L. L. Hu Y. Zhang P. Zhang Z. Li L. H. Gao G. D. . (2018). Neural stem cell transplantation promotes functional recovery from traumatic brain injury via brain derived neurotrophic factor-mediated neuroplasticity. Mol. Neurobiol. 55, 2696–2711. doi: 10.1007/s12035-017-0551-1 28421542

[B92] Xiong Y. Mahmood A. Chopp M. (2024). Mesenchymal stem cell-derived extracellular vesicles as a cell-free therapy for traumatic brain injury via neuroprotection and neurorestoration. Neural Regen. Res. 19, 49–54. doi: 10.4103/1673-5374.374143 37488843 PMC10479856

[B93] Xu G. Jin J. Fu Z. Wang G. Lei X. Xu J. . (2025). Extracellular vesicle-based drug overview: research landscape, quality control and nonclinical evaluation strategies. Signal Transduct. Target. Ther. 10:255. doi: 10.1038/s41392-025-02312-w 40804047 PMC12350758

[B94] Yang Y. Wei H. Zhou X. Zhang F. Wang C. (2017). Hyperbaric oxygen promotes neural stem cell proliferation by activating vascular endothelial growth factor/extracellular signal-regulated kinase signaling after traumatic brain injury. Neuroreport 28, 1232–1238. doi: 10.1097/WNR.0000000000000901 28953090

[B95] Ye Y. Xu H. Zhang X. Li Z. Jia Y. He X. . (2014). Association between toll-like receptor 4 expression and neural stem cell proliferation in the hippocampus following traumatic brain injury in mice. Int. J. Mol. Sci. 15, 12651–12664. doi: 10.3390/ijms150712651 25036030 PMC4139865

[B96] Yuan F. Y. Zhang M. X. Shi Y. H. Li M. H. Ou J. Y. Bai W. F. . (2020). Bone marrow stromal cells-derived exosomes target DAB2IP to induce microglial cell autophagy, a new strategy for neural stem cell transplantation in brain injury. Exp. Ther. Med. 20, 2752–2764. doi: 10.3892/etm.2020.9008 32765770 PMC7401953

[B97] Zamproni L. N. Mundim M. V. Porcionatto M. A. des Rieux A. (2017). Injection of SDF-1 loaded nanoparticles following traumatic brain injury stimulates neural stem cell recruitment. Int. J. Pharm. 519, 323–331. doi: 10.1016/j.ijpharm.2017.01.036 28115261

[B98] Zhang K. Jiang Y. Wang B. Li T. Shang D. Zhang X. . (2022). Mesenchymal stem cell therapy: a potential treatment targeting pathological manifestations of traumatic brain injury. Oxid. Med. Cell. Longev. 2022:4645021. doi: 10.1155/2022/4645021 35757508 PMC9217616

[B99] Zhang L. Bai W. Peng Y. Lin Y. Tian M. (2024). Human umbilical cord mesenchymal stem cell-derived exosomes provide neuroprotection in traumatic brain injury through the lncRNA TUBB6/Nrf2 pathway. Brain Res. 1824:148689. doi: 10.1016/j.brainres.2023.148689 38030103

[B100] Zhang L. Lin Y. Bai W. Sun L. Tian M. (2023a). Human umbilical cord mesenchymal stem cell-derived exosome suppresses programmed cell death in traumatic brain injury via PINK1/Parkin-mediated mitophagy. CNS Neurosci. Ther. 29, 2236–2258. doi: 10.1111/cns.14159 36890626 PMC10352888

[B101] Zhang R. Liu Y. Yan K. Chen L. Chen X. R. Li P. . (2013). Anti-inflammatory and immunomodulatory mechanisms of mesenchymal stem cell transplantation in experimental traumatic brain injury. J. Neuroinflammation 10:106. doi: 10.1186/1742-2094-10-106 23971414 PMC3765323

[B102] Zhang X. Hei Y. Bai W. Huang T. Kang E. Chen H. . (2020a). Toll-like receptor 2 attenuates traumatic brain injury-induced neural stem cell proliferation in dentate gyrus of rats. Neural Plast. 2020:9814978. doi: 10.1155/2020/9814978 32879625 PMC7448220

[B103] Zhang X. Wang J. Lu Y. Xu F. Cheng X. Qin J. . (2026). YTHDF1-modified neural stem cells confer neuroprotection and promote functional recovery following traumatic brain injury. Brain Res. Bull. 243:112015. doi: 10.1016/j.brainresbull.2026.112015 42336160

[B104] Zhang Y. Liu J. Zhou Y. Zou Z. Xie C. Ma L. . (2023b). miR-18a-5p shuttled by mesenchymal stem cell-derived extracellular vesicles alleviates early brain injury following subarachnoid hemorrhage through blockade of the ENC1/p62 axis. Cell Tissue Res. 392, 671–687. doi: 10.1007/s00441-023-03754-w 36795153

[B105] Zhang Y. Zhang Y. Chopp M. Zhang Z. G. Mahmood A. Xiong Y. . (2020b). Mesenchymal stem cell-derived exosomes improve functional recovery in rats after traumatic brain injury: a dose-response and therapeutic window study. Neurorehabil. Neural Repair 34, 616–626. doi: 10.1177/1545968320926164 32462980 PMC7329589

[B106] Zheng Y. Wu G. Chen L. Zhang Y. Luo Y. Zheng Y. . (2021). Neuro-regenerative imidazole-functionalized GelMA hydrogel loaded with hAMSC and SDF-1alpha promote stem cell differentiation and repair focal brain injury. Bioact. Mater. 6, 627–637. doi: 10.1016/j.bioactmat.2020.08.026 33005827 PMC7508914

[B107] Zhong L. Wang J. Wang P. Liu X. Liu P. Cheng X. . (2023). Neural stem cell-derived exosomes and regeneration: cell-free therapeutic strategies for traumatic brain injury. Stem Cell Res. Ther. 14:198. doi: 10.1186/s13287-023-03409-1 37553595 PMC10408078

[B108] Zhou H. X. Liu Z. G. Liu X. J. Chen Q. X. (2016). Umbilical cord-derived mesenchymal stem cell transplantation combined with hyperbaric oxygen treatment for repair of traumatic brain injury. Neural Regen. Res. 11, 107–113. doi: 10.4103/1673-5374.175054 26981097 PMC4774201

[B109] Zhou Y. Shao A. Xu W. Wu H. Deng Y. (2019). Advance of stem cell treatment for traumatic brain injury. Front. Cell. Neurosci. 13:301. doi: 10.3389/fncel.2019.00301 31456663 PMC6700304

